# Understanding leptospirosis eco-epidemiology by environmental DNA metabarcoding of irrigation water from two agro-ecological regions of Sri Lanka

**DOI:** 10.1371/journal.pntd.0008437

**Published:** 2020-07-23

**Authors:** Chandika D. Gamage, Yukuto Sato, Ryosuke Kimura, Tetsu Yamashiro, Claudia Toma

**Affiliations:** 1 Department of Microbiology, Faculty of Medicine, University of Peradeniya, Peradeniya, Kandy, Sri Lanka; 2 Center for Strategic Research Project, Organization for Research Promotion, University of the Ryukyus, Nishihara, Okinawa, Japan; 3 Department of Human Biology and Anatomy, Graduate School of Medicine, University of the Ryukyus, Nishihara, Okinawa, Japan; 4 Department of Bacteriology, Graduate School of Medicine, University of the Ryukyus, Nishihara, Okinawa, Japan; Universidade Federal de Pelotas, BRAZIL

## Abstract

**Background:**

Leptospirosis is one of the most significant zoonoses across the world not only because of its impact on human and animal health but also because of the economic and social impact on agrarian communities. Leptospirosis is endemic in Sri Lanka where paddy farming activities, the use of draught animals in agriculture, and peridomestic animals in urban and rural areas play important roles in maintaining the infection cycle of pathogenic *Leptospira*, especially concerning animals as a potential reservoir. In this study, an environmental DNA (eDNA) metabarcoding methodology was applied in two different agro-ecological regions of Sri Lanka to understand the eco-epidemiology of leptospirosis.

**Methodology/Principal findings:**

Irrigation water samples were collected in Kandy District (wet zone mid-country region 2) and Girandurukotte, Badulla District (intermediate zone low-country region 2); and analysed for the presence of pathogenic *Leptospira*, associated microbiome and the potential reservoir animals. Briefly, we generated PCR products for high-throughput sequencing of multiple amplicons through next-generation sequencing. The analysis of eDNA showed different environmental microbiomes in both regions and a higher diversity of *Leptospira* species circulating in Kandy than in Girandurukotte. Moreover, the number of sequence reads of pathogenic *Leptospira* species associated with clinical cases such as *L*. *interrogans* was higher in Kandy than in Girandurukotte. Kandy also showed more animal species associated with pathogenic bacterial species than Girandurukotte. Finally, several pathogenic bacterial species including *Arcobacter cryaerophilus*, responsible for abortion in animals, was shown to be associated with pathogenic *Leptospira*.

**Conclusions/Significance:**

Leptospirosis has been considered to be endemic in wet regions, consistently, leptospiral sequences were detected strongly in Kandy. The great *Leptospira* species diversity in Kandy observed in this study shows that the etiological agents of leptospirosis in Sri Lanka might be underestimated. Furthermore, our eDNA metabarcoding can be used to discriminate bacterial and animal species diversity in different regions and to explore environmental microbiomes to identify other associated bacterial pathogens in the environment.

## Author summary

Leptospirosis is a widespread bacterial zoonosis with increasing importance due to its vast range of reservoir hosts. Early symptoms are shared by other infectious diseases common in tropical and sub-tropical regions, where the real burden and risk factors need to be known. In Sri Lanka, leptospirosis is mostly an occupational disease associated with freshwater or animal exposure in agriculture communities. Thus, there is a need for understanding the epidemiology of leptospirosis in agrarian regions of the country for developing better prevention and intervention strategies. In this study, we applied an environmental DNA metabarcoding methodology to understand the environmental microbiome, potential reservoir animals and the *Leptospira* species circulating in two different agro-ecological regions of Sri Lanka: Kandy (wet region mid-country region 2) and Girandurukotte (intermediate region low-country region 2). It is known that pathogenic *Leptospira* are excreted through the urine of reservoir animals in the environment, where they can persist in humid conditions. Congruently, this study showed a higher detection of pathogenic *Leptospira* in the environment of Kandy where the environmental microbiome showed a higher diversity than Girandurukotte. Potential animal reservoirs were also detected in samples positive for pathogenic *Leptospira*, suggesting that environmental DNA metabarcoding can provide important information for management and intervention strategies to control leptospirosis.

## Introduction

*Leptospira* is a bacterial genus which comprises 64 species classified into 4 subclades, called P1, P2, S1 and S2; historically named as pathogens (P1), intermediates (P2) and saprophytes (S1 and S2) [1]. Subclade P1 can be further split into “virulent” and “low virulence” pathogens based on phylogenomic analysis and the outcome in patients and/or virulence in animal models [2]. Virulent pathogens species (*L*. *interrogans*, *L*. *borgpetersenii*, *L*. *kirschneri*, *L*. *noguchii*, among others*)* are the main agents of leptospirosis, a worldwide zoonotic disease with increasing importance due to the vast range of reservoir hosts and climate change [2, 3]. Low-virulence pathogens such as *L*. *kmetyi* are frequently isolated from the environment but their virulence potential is still controversial [1, 2].

Chronically infected reservoir animals excrete live, fully virulent leptospires in their urine and contaminate the environment both soil and surface water sources. Pathogenic leptospires can survive in the environment for a couple of weeks to several months, depending on the climatic factors and nature of the surface water, and soil texture [4–7]. Humans get exposure to the pathogenic leptospires either through direct contact with the urine of chronically infected domestic or wild animals, or indirectly through contaminated environmental sources during outdoor activities such as swimming or occupation-related activities including preparing paddy lands, harvesting of rice, and handling of livestock and pet animals [4]. Thus, exposure to environmental pathogenic leptospires varies greatly depending on each geographical region [8]. In order to understand *Leptospira* transmission cycle, pathogenic *Leptospira* species from several environmental sources were successfully isolated using a novel combination of antimicrobial agents. However, few reports have successfully isolated clinically important species such as *L*. *interrogans*, belonging to the pathogenic P1 group [9–13].

Sri Lanka is a tropical island divided into three zones based on elevation: low-country (0–300 m), mid-country (300–900 m), and up-country (>900 m). The annual rainfall is not uniform all over the island and the country is divided into three major climatic zones on the basis of total rainfall: wet zone (2500–5500 mm annual rainfall), intermediate zone (1750–2500 mm annual rainfall with a short dry season), and dry zone (below 1750 mm annual rainfall). The island is composed of 13 major soil groups. Thus, the climatic zones have been further subdivided into agro-ecological regions according to a particular combination of climate, soil, and terrain conditions [14]. Leptospirosis is a common cause of febrile illness, mainly from agro-ecological regions of the wet zone where the pathogenic cycle is favoured by paddy farming activities, high rainfall, moist soil, and the use of buffalo in agriculture [15]. However, an outbreak of leptospirosis was reported in a relatively dry district in 2011 and the true burden of leptospirosis is supposed to be underestimated in the national statistics due to the lack of point of care diagnostic tests [4, 16]. Moreover, the global climate change will increase evapotranspiration and soil moisture deficit in dry zone areas, demanding irrigation water to compensate the crop water requirement. In contrast, in wet zones, more floods will increase the risk of leptospirosis. According to a hospital-based sentinel surveillance of the Ministry of Health Care, Sri Lanka, almost 80% of suspected leptospirosis patients have had exposure to paddy and marshy lands before developing disease symptoms, and almost 60% of these were farmers [17]. Furthermore, a case-control study of environmental and occupational risks of leptospirosis in Sri Lanka also showed that paddy workers have a higher odds ratio of leptospirosis transmission than non-paddy workers [18]. Most small-holder dairy farmers allow their cattle to graze in paddy fields before the cultivation starts, thus, household animals seemed to be important for transmission of leptospirosis to humans [18]. In Sri Lanka, cattle demonstrate a high seropositive rate for leptospirosis [19, 20]. Furthermore, pathogenic *Leptospira* have been detected either by isolation or molecular techniques in a wide range of reservoir animals such as bandicoots, rats, dogs, and domesticated elephants [21, 22]. A meta-analysis to estimate the load of *Leptospira* excreted in urine had also shown that large mammals which excrete much more urine than rats shed significantly more *Leptospira* per day [23]. Thus, in agrarian communities, large animals such as buffalo and cattle might be the main source excreting pathogenic leptospires into paddy lands.

Environmental DNA (eDNA) in aquatic environments originates from various sources, including feces, urine, damaged tissue, and microorganisms, and has successfully been used to identify pathogenic *Leptospira* and associated organisms in rivers of leptospirosis endemic areas of Japan [24]. In paddy lands, irrigation water can be a reservoir and source of spreading urine-containing *Leptospira*. The role of irrigation water in the maintenance of infectious *Leptospira* needs to be analyzed since urban ornamental water fountains exposed to rodents and stray animals have been recently suggested to act as temporary carriers of pathogenic *Leptospira* [25]. Bacteria excreted into the environment are able to form biofilms by interacting with other organisms which might be a strategy to maintain a sufficiently concentrated amount of bacteria in order to achieve infection [26, 27]. The composition of microbial communities, which is dependent on the climate and soil properties of each agro-ecological region, also influences the formation of biofilms, and thus, the persistence of infectious *Leptospira* in the environment. The etiological agents of leptospirosis in Sri Lanka belong to species of the P1 subclade/virulent group (*L*. *interrogans*, *L*. *kirschneri*, *L*. *borgpetersenii*, *L*. *santorasai and L*. *weilii*) [22, 28]. Little to no information is available regarding the environmental *Leptospira* species of Sri Lanka.

The identification of the pathogenic *Leptospira* reservoir animals in the different agro-ecological regions of Sri Lanka will be useful to understand the eco-epidemiology of leptospirosis and establish proper prevention and intervention strategies. In this study, we applied an eDNA metabarcoding approach to compare the microbiome and species diversity of environmental leptospires in two different agro-ecological regions of Sri Lanka to understand how the different climate, soil and terrains conditions influence the environmental transmission of pathogenic *Leptospira*.

## Methods

### Environmental water sampling

**Fig 1:**
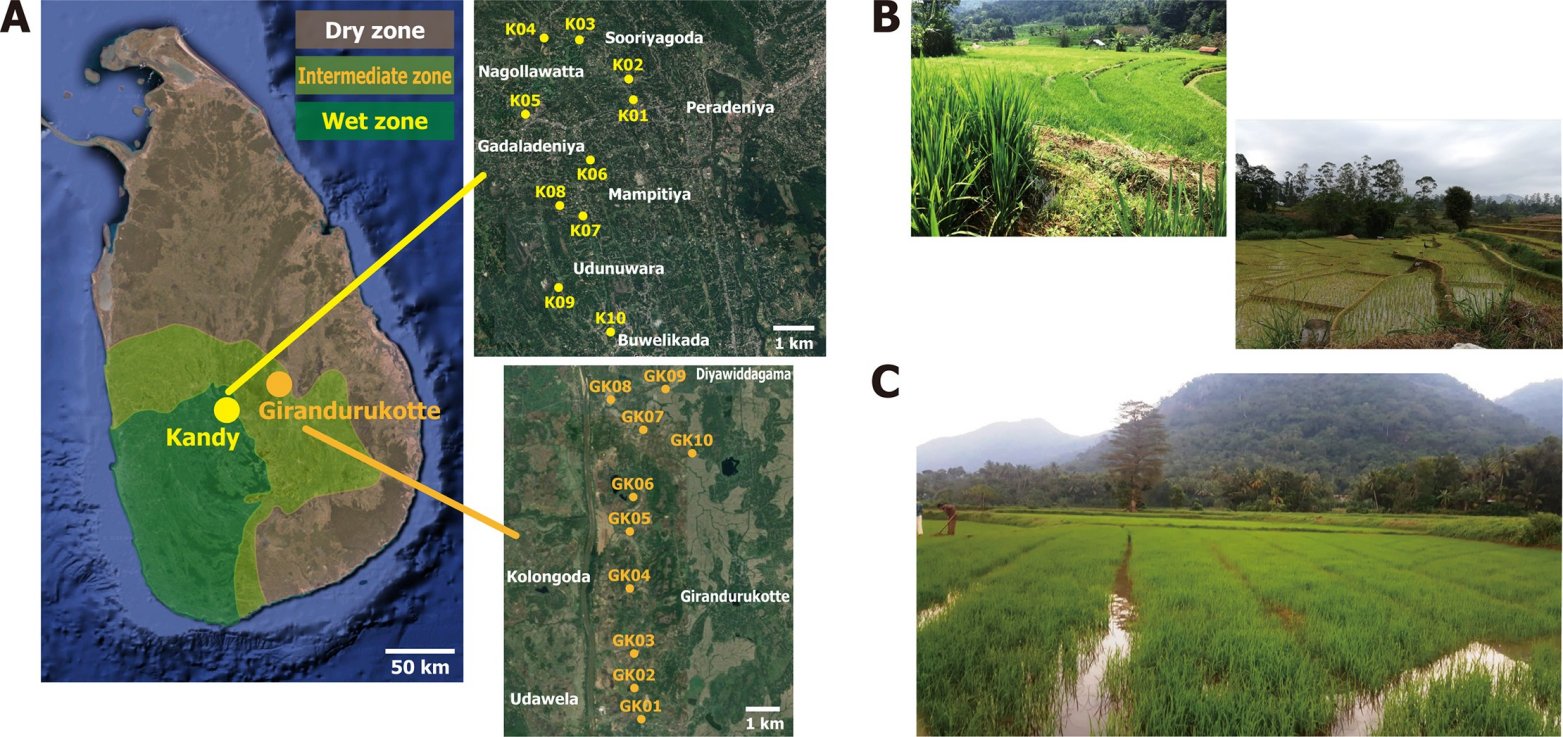
Sampling locations of environmental water. **(**A) Representative agro-ecological regions of Sri Lanka are shown by color shading: dark green, light green, and brown indicating wet, intermediate, and dry zones, respectively. K01 to K10, Kandy sampling sites; and GK01 to GK10, Girandurukotte sampling sites, respectively. Satellite imagery were obtained from Google Maps (https://www.google.com/maps/); data providers of the satellite imagery are Google, Data SIO, NOAA, U.S. Navy, NGA, GEBCO, Maxar Technologies, CNES/Airbus, Landsat/Copernicus, and TerraMetrics. Adobe Illustrator CS6 was used to create the map with satellite imagery. B) Representative landscape of paddy fields in a mountainous region of Kandy. C) Representative landscape of a paddy field in a flat region of Girandurukotte.

Irrigation water sampling was conducted in two representative agro-ecological regions in the central region of Sri Lanka: Yatinuwara and Udunuwara Divisional Secretariats (DS), Kandy District (wet zone mid-country region 2), and Girandurukotte, Mahiyanganaya DS, Badulla District (intermediate zone low-country region 2). Kandy District belongs to wet zone where average annual rainfall is above 2000 mm throughout the year and average annual temperature is 25°C. Badulla District belongs to intermediate zone, annual rainfall is around 1800 mm mainly from October to December, and average annual temperature is around 23°C. Both districts have a similar population density of cattle and buffalo [29], while human population density is higher for Kandy District (709 people/km^2^) than Badulla District (285 people/km^2^) [30]. Water samples were collected at ten locations in each region (K01−K10 and GK01−GK10 in Fig 1A) selected according to notified cases of leptospirosis informed by Public Health Inspectors of both Districts. In each location, irrigation water was manually collected at the agricultural or rice fields by wearing disposable gloves, using a clean plastic ladle, and 500 mL to 1,000 mL water was filtrated by the Sterivex filter with 0.45 μm pore size membrane (Merck Millipore, Milan, Italy), capable to capture leptospiral cells [24], using a 60 mL disposable syringe. After filtration, the filter unit was filled with 2 mL of DNAiso Reagent (Takara, Shiga, Japan) and tightly sealed by polypropylene luer-fitting caps (ISIS, Osaka, Japan) until DNA extraction was carried out at room temperature. The water sampling was conducted on 20 December, 2018 in Kandy (collectively Udunuwara and Yatinuwara DSs) and 26 December, 2018 in Girandurukotte, respectively.

### DNA extraction

Environmental DNA was extracted from the Sterivex filters by using the DNeasy PowerWater Sterivex Kit (Qiagen, Hilden, Germany) according to a standard protocol with a slight modification as given below. Before the DNA extraction, the DNAiso Reagent was ejected from filter units using a 10 mL disposable syringe. After the addition of incubated MBL Solution to the filters, the filters were further incubated at 65°C on a heat block for 10 minutes to lyse cells and to denaturate proteins in the filtrated residues. In a final elution step of DNA, 25 μL of Microbial DNA-Free Water (Qiagen) was used to avoid unexpected contamination from common elution buffers and reagents. The elution was conducted two times, obtaining a total volume of 50 μL of eDNA solution from each filter unit. The eDNA samples were stored at −30°C after verification of the DNA concentration and quality (OD_260/280_ >1.80) using Nanodrop 2000c Spectrophotometer (Thermo Fisher Scientific, Waltham, USA).

### PCR amplification for metabarcoding sequencing of *Leptospira* and other bacteria

A two-step, tailed PCR method was applied to amplify partial fragments of the 16S rRNA and of *lipL32* genes of *Leptospira*, and those of 16S rRNA V4 region of broad bacteria from the prepared eDNA samples as described previously [24]. The PCR primers for the targeted region of *Leptospira* 16S rRNA [31], *lipL32* [32], and universal bacterial 16S rRNA genes [33] were used along with additional MiSeq sequencing priming sites and random hexamer nucleotides to improve the base-call calibration of the MiSeq platform (Illumina, San Diego, CA, USA) (Table 1). The typical length of the target region of leptospiral 16S rRNA, *lipL32*, and bacterial 16S rRNA V4 was 330, 242, and 259 base pairs (bp), respectively. The final concentration of the primer pair was 0.38 μM for leptospiral 16S rRNA and 0.19 μM for leptospiral *lipL32* and bacterial 16S rRNA, respectively. The PCR was performed in a multiplex manner using Multiplex PCR Assay Kit version 2 (Takara, Shiga, Japan) with 2.0 μL of template eDNA in a total reaction volume of 10.0 μL through 37 cycles of thermal cycle profiles, as previously described [24]. The first-round PCR products were diluted 30-fold in RNase-free water (Thermo Fisher Scientific/Invitrogen, Carlsbad, USA) and subjected to second-step indexing PCR to add the dual-index tags (D5 and D7 series; Illumina) and MiSeq flowcell binding site sequences by using Ex Taq Hot Start (HS) Version (Takara) [24, 33].

**Table 1:** Tailed PCR primers used for environmental DNA metabarcoding sequencing by Illumina MiSeq platform in this study.

Primer names	PCR targets^1^	Sequence (5'–3')^2^^,^^3^	References
Lepat 1	Lepto 16S rRNA	ACACTCTTTCCCTACACGACGCTCTTCCGATCT NNNNNNGAGTCTGGGATAACTTT	[31]
Lepat 2	Lepto 16SrRNA	GTGACTGGAGTTCAGACGTGTGCTCTTCCGATCT NNTCACATCGYTGCTTATTTT	[31]
LipL32- 45F	*lipL32*	ACACTCTTTCCCTACACGACGCTCTTCCGATCTNN NNNNAAGCATTACCGCTTGTGGTG	[32]
LipL32- 286R	*lipL32*	GTGACTGGAGTTCAGACGTGTGCTCTTCCGATCTN NGAACTCCCATTTCAGCGATT	[32]
16S rRNA V4 F	Bacterial 16SrRNA	ACACTCTTTCCCTACACGACGCTCTTCCGATCTNN NNNNGTGCCAGCMGCCGCGGTAA	[33]
16S rRNA V4 R	Bacterial 16SrRNA	GTGACTGGAGTTCAGACGTGTGCTCTTCCGATCTN NNNNNGGACTACHVGGGTWTCTAAT	[33]
L-flaB-F1	*flaB*	ACACTCTTTCCCTACACGACGCTCTTCCGATCTNNN NNNCTCACCGTTCTCTAAAGTTCAAC	[34]
L-flaB-R1	*flaB*	GTGACTGGAGTTCAGACGTGTGCTCTTCCGATCTNN NNNNTGAATTCGGTTTCATATTTGCC	[34]
L-flaB-F2	*flaB* (nested)	ACACTCTTTCCCTACACGACGCTCTTCCGATCTNNN NNNTGTGCACAAGACGATGAAAGC	[35]
L-flaB-R2	*flaB* (nested)	GTGACTGGAGTTCAGACGTGTGCTCTTCCGATCTNN NNNNAACATTGCCGTACCACTCTG	[35]
MiFish-U-F	Vertebrate 12S rRNA	ACACTCTTTCCCTACACGACGCTCTTCCGATCTNNN NNNGTCGGTAAAACTCGTGCCAGC	[36]
MiFish-U-R	Vertebrate 12S rRNA	GTGACTGGAGTTCAGACGTGTGCTCTTCCGATCTNN NNNNCATAGTGGGGTATCTAATCCCAGTTTG	[36]

^1^PCR targets are partial gene fragments.

^2^Positions with mixed bases are designated by their IUB nucleotide codes: R = A/G; Y = C/T; K = G/T; M = A/C; S = G/C; W = A/T; N = A/G /C/T.

^3^Target-specific nucleotides are underlined.

### PCR amplification and sequencing of partial *flaB* gene of pathogenic *Leptospira*

For better taxonomic resolution of pathogenic *Leptospira* species than that of partial 16S rRNA gene, we also performed nested PCR amplification and sequencing of partial *flaB* gene [34, 35]. PCR primers L-flaB-F1 and L-flaB-R1 were used for first-round PCR, and L-flaB-F2 and L-flaB-R2 were used for second-round PCR with additional MiSeq sequencing priming sites and random hexamer nucleotides for sequencing purposes as described earlier [33]. The typical length of the target region of 2nd-round PCR product was 576 bp. These two rounds of PCR were performed using PrimeSTAR HS DNA Polymerase (TaKaRa) with 0.30 μM of each primer and a total reaction volume of 10.0 μL. In the 1st-round PCR, 1.5 μL of template eDNA was used and the thermal cycle profile was as follows: 94°C for 3 min followed by 30 cycles at 98°C for 10 sec, 50°C (replicate 1) or 55°C (replicate 2) for 15 sec, and 72°C for 30 sec with a final extension at 72°C for 5 min. In the 2nd-round PCR, 1.5 μL of 1st-round PCR product was used as template and thermal cycle profile was as follows: 94°C for 3 min followed by 30 cycles at 98°C for 10 sec, 63°C for 15 sec, and 72°C for 30 sec with a final extension at 72°C for 5 min. The 2nd-round PCR product was diluted 50-fold in RNase-free water (Thermo Fisher Scientific/Invitrogen) and subjected to indexing PCR to add the dual-index tags (D5 and D7 series; Illumina) and MiSeq flowcell binding site sequences by using Ex Taq Hot Start (HS) Version (Takara) as described earlier [24, 33].

### PCR amplification for metabarcoding sequencing of vertebrates

A two-step, tailed PCR was also carried out to perform eDNA metabarcoding analysis of vertebrate animals in order to assess the potential host organisms of *Leptospira* by using MiFish primers [36] as described previously [24]. In the first step PCR, typically 169-bp fragment of vertebrate mitochondrial (mt) DNA 12S rRNA was amplified by using two PCR enzymes with two different annealing temperatures each: HiFi HotStart ReadyMix (KAPA Biosystems, Wilmington, MA USA) at annealing temperatures of 60 and 65°C and PrimeSTAR HS DNA polymerase (Takara) at annealing temperatures of 50 and 55°C. The PCR conditions including primer concentration and thermal cycle profile were the same as previously described [24]. In the second step PCR, the first-round products were diluted 20-fold with RNase-free water (Thermofisher Scientific/Invitrogen), and dual-index tags and flowcell binding sites were added by using Ex Taq HS Version (Takara) [24, 33].

### DNA sequencing of metabarcoding PCR products

The tag-indexed second-round PCR products were sequenced by Illumina MiSeq platform with V2 chemistry. The PCR products with unique combinations of dual-index tags were pooled in equal amounts for semi-quantitative analysis and purified by using the 1.5% L03 agarose gel (Takara) and a MinElute Gel Extraction Kit (Qiagen) according to a standard protocol. The eluted DNA solution was further purified using a 1.8-fold amount of AMPure XP magnetic beads (Beckman Coulter, High Wycombe, UK) with a standard purification protocol using 70% ethanol. The obtained sequencing library was quantified using Qubit 2.0 and dsDNA HS Assay Kit (Thermo Fisher Scientific). The 30 pM-library was subjected to DNA sequencing using MiSeq Reagent Kit V2 (Illumina). The 250 and 150 bp paired-end sequencing was adapted to multiplex leptospiral/bacterial and MiFish vertebrate mitochondrial library, respectively. The volume molarity was calculated based on the averaged molecular weight of a nucleotide (ca. 660 g per 1 bp), DNA concentration of the sequencing library, and the mean target length (bp) of second-round PCR products in the library.

### Sequence data analysis of *Leptospira*, bacteria, and vertebrate metabarcoding

Raw read data sequenced by the MiSeq was subjected to primary processing based on the sequence data quality. The low-quality 3′-tail of each sequence with >10^−1^ error rate was removed using the program DynamicTrim provided in the SolexaQA software package [37]. The tail-trimmed paired-end sequences were connected by the software FLASH [38] and processed by custom Perl scripts to remove reads containing basecall failures (N bases) and showing atypical length compared with expected sizes of the PCR products described earlier. Exceptionally the paired-end sequences of the *flaB* gene were not connected because the target region length (576 bp) is longer and thus the paired reads do not wrap over with each other. Alternatively, the forward and reverse reads of *flaB* were concatenated in 5'-to-3' order in this step. Primer sequences were searched and removed using TagCleaner [39] with a maximum five-base mismatch. The sequences without primers at either end were excluded in this step. Finally, we merged redundant reads of each sample into one de-replicated sequence while keeping the count information intact using UCLUST (derep_fulllength command) [40]. In this step, singleton reads in each sample lacking reproducibility were excluded.

The quality-filtered, effective sequences were analyzed to estimate their taxonomic origin based on the sequence similarity with known reference sequences by using the NCBI (National Center for Biotechnology Information, USA) BLAST plus program [41]. As reference database, the National Center for Biotechnology Information (NCBI) nucleotide (nt) [42] was used for the analysis of leptospiral 16S rRNA, *lipL32*, and *flaB* genes; the GreenGenes [43] for broad bacterial 16S rRNA gene V4 region; and the MitoFish [24, 44] and the NCBI nt for vertebrate mt 12S rRNA genes.

For the leptospiral 16S rRNA and *flaB* sequences, the similarity-based annotations were further corrected carefully via molecular phylogeny. The Blastn analysis was performed at similarity and *e*-value thresholds of 85% and 10^−3^, respectively, to avoid false negative (type II) errors in this primary sequence annotation. The provisional annotations according to the obtained Blast top hits were confirmed or corrected based on phylogenetic analysis with known sequences of representative *Leptospira* species [1]. Multiple sequence alignments were performed by MAFFT [45], and maximum-likelihood phylogenetic tree was estimated using MEGA version 7.0.14 [46]. A general time-reversible model of nucleotide substitution [47] was applied with auto adjustment of invariable site and gamma correction parameters. For the analysis of bacterial 16S rRNA gene V4 region, sequences that appeared more than 10 times in at least one sample were used for further analysis to eliminate lower count-reads of potential PCR chimeras and sequencing errors. The Blastn-based species annotation was conducted with sequence similarity and *e*-value thresholds of the same values as those for leptospiral sequences.

For the vertebrate mt 12S rRNA genes, re-mapping analysis of once excluded singleton reads were performed to absorb possible sequencing errors. The singletons were re-aligned to remaining sequences with ≥2 read counts at 99% sequence similarity threshold, and the number of aligned singletons was added to the count information of the matched subject sequence. Unmapped singletons were discarded in this step. These reliable sequences with corrected count information were then subjected to the Blastn-based species annotation at increased sequence-similarity and *e*-value thresholds of 97% and 10^−5^, respectively, because the database completeness of vertebrate mt DNA was in general higher than that for bacteria and *Leptospira*. The sequence counts of PCR replicates were summed up for each sample.

Multivariate analyses were performed by using the software programs PAST [48] and R (http://www.r-project.org/). In all of the correlation analysis of the present study, we applied a simple Pearson's product moment correlation coefficient (*r*) for the comparisons of sequence read numbers among *Leptospira*, bacteria, and vertebrates. The two-sided significance level of 0.05 was adapted and corrected false discovery rates were estimated based on the Benjamini–Hochberg method for multiple testing. Partial correlations were calculated using the R script named pcor.R [49].

### Accession numbers

Raw reads generated during the current study are available in the DDBJ Sequence Read Archive (DRA) under the accession numbers: DRA009844, DRA009845, and DRA009846.

## Results

### Environmental DNA collection and multiplex metabarcoding sequencing of *Leptospira* in Sri Lanka

#### DNA sampling and extraction results

To examine the eDNA-based approach for detection of pathogenic *Leptospira*, *Leptospira*-associated environmental bacteria, and potential reservoir animals in Sri Lanka, a total of twenty eDNA samples were collected in two representative agro-ecological regions of the Sri Lanka Island, Kandy (K) and Girandurukotte (GK) (Fig 1A). We extracted highly pure eDNA in eighteen samples with average concentration and OD_260/280_ values of 3.9 ± 0.6 ng/μL (mean ± S.E.) and 3.3 ± 0.5 (S1 Table). Exceptionally, high concentrations were observed in eDNA of GK07 and GK08 (47.0 and 168.7 ng/μL, respectively) potentially due to contamination by large soil particles or organismal fragments into the filter units. All of these twenty samples were subjected to following PCR experiments without dilution for semi-quantitative purpose.

#### Sequencing results

From each eDNA sample, partial fragments of leptospiral 16S rRNA [31] and *lipL32* [32] genes and broad bacterial 16S rRNA gene V4 region [33] were amplified by multiplex PCR. PCR was replicated twice for each sample and independently sequenced, producing in total 3,611,521 pairs of raw sequences with 90,288 ± 2,313 reads per a replicate (DRA009844). After quality-based processing of the data, a total of 3,336,072 sequences remained as quality-filtered ones, with 83,402 ± 2,197 sequences per a replicate. Among them, both primer ends of the bacterial 16S rRNA V4 were found in 3,268,705 sequences (81,718 ± 2,193 reads per a replicate), those of leptospiral 16S rRNA were found in 19,673 sequences (492 ± 171 reads per a replicate), and those of *lipL32* were found in 47,694 sequences (1,192 ± 214 reads per a replicate).

### Analysis of environmental leptospiral DNA sequences particularly focusing on pathogenic species

#### 16S rRNA sequences analysis

**Fig 2:**
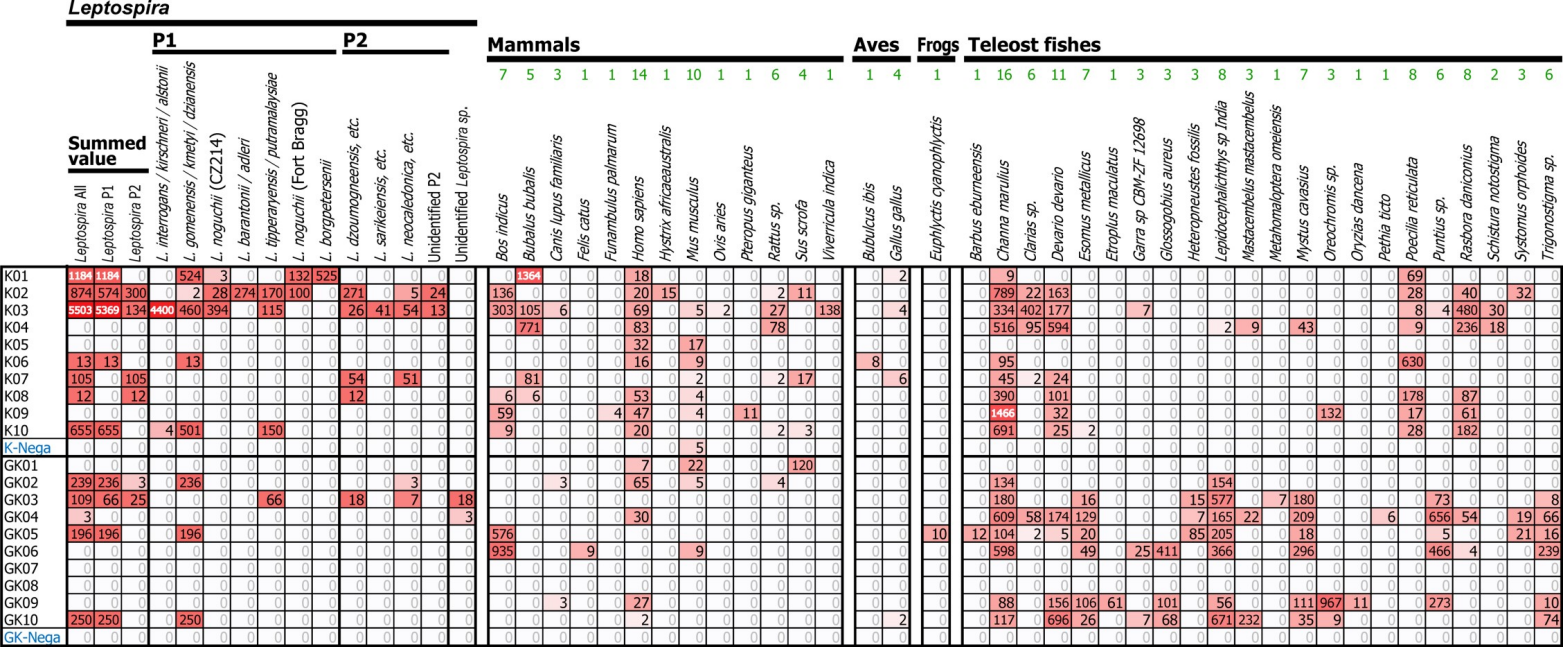
Environmental detection of leptospiral 16S rRNA gene and vertebrate mitochondrial 12S rRNA gene. The number of sequence reads detected in each sample are shown with colored matrices in red shading, where the red color intensity is relative to the number of sequence reads. Color gradation from light red to dark red represents low number to high number of sequence reads, respectively. More than thousand sequence reads are denoted in dark red cells and white bold numbers. P1 and P2 denote phylogenetic subclades of *Leptospira*. Green numbers above the scientific name of animals indicate the number of times of appearance of the species in total of 20 water samples. K and GK indicate sampling locations Kandy and Girandurukotte, respectively. K01−K10 and GK01−GK10 denote sample names. K-nega and GK-nega indicate negative control samples (RNase free water).

From the filtered 19,673 sequences of leptospiral 16S rRNA gene, we found a total of 9,143 non-singleton (≥2 counts), effective sequences (red shading in a panel denoted as *Leptospira* in Fig 2). The remaining sequences were singletons in a sample (in total 8,478 sequences; on average 212 ± 71 in a replicate) or sequences with no hits (in total 2,040 sequences) or those with a hit to other unknown bacteria (in total 12 sequences) in the BLAST database. The species annotation of the sequences was corrected based on a molecular phylogenetic analysis using known sequences of the *Leptospira* 16S rRNA gene and their latest nomenclature [1] (S1 Fig). Leptospiral 16S rRNA sequences, which included reads from the P1-virulent group (e.g., *L*. *interrogans/L*. *kirschneri/L*. *alstonii*, etc.), were detected numerously in the K03 sample from Kandy (dark red shading in Fig 2; 5,503 reads in total). Similarly, relatively strong signals were detected in other K samples (*e*.*g*., 1,184 sequences in K01, 874 in K02, and 655 in K10), which included putative signals of *L*. *interrogans* and other pathogenic *Leptospira* of P1-virulent group (e.g., *L*. *noguchii*, *L*. *borgpetersenii*, etc.), and also species from P2 subclade (*e*.*g*., *L*. *dzoumogneensis*, *L*. *sarikeiensis*, etc.). On the other hand, relatively smaller counts of leptospiral 16S rRNA sequences were detected in GK samples (*e*.*g*., a maximum of 250 sequences from GK10), although the species from P1-low virulence group and P2 subclades were also detected (*e*.*g*., *L*. *gomenensis* and *L*. *dzoumogneensis*).

#### *lipL32* sequences analysis

From the 47,694 putative *lipL32* sequences, we obtained a total of 32,520 non-singleton, effective sequences. Among them, in total 5,609 were the true-positive sequences of leptospiral *lipL32* gene, while other sequences indicated BLAST hits to other bacteria (total 666 sequences) or no database hit (total 26,245 sequences; S2 Fig). From this *lipL32* gene result, pathogenic *L*. *interrogans* was detected again in K03 sample similar to the results of leptospiral 16S rRNA. There were only a few samples with detection of *Leptospira* (putative *L*. *kmetyi* in K02 and unknown *Leptospira sp*. in K10) and no detection in GK samples, indicating relatively low sensitivity and specificity of this leptospiral *lipL32* analysis.

#### *flaB* sequences analysis

**Fig 3:**
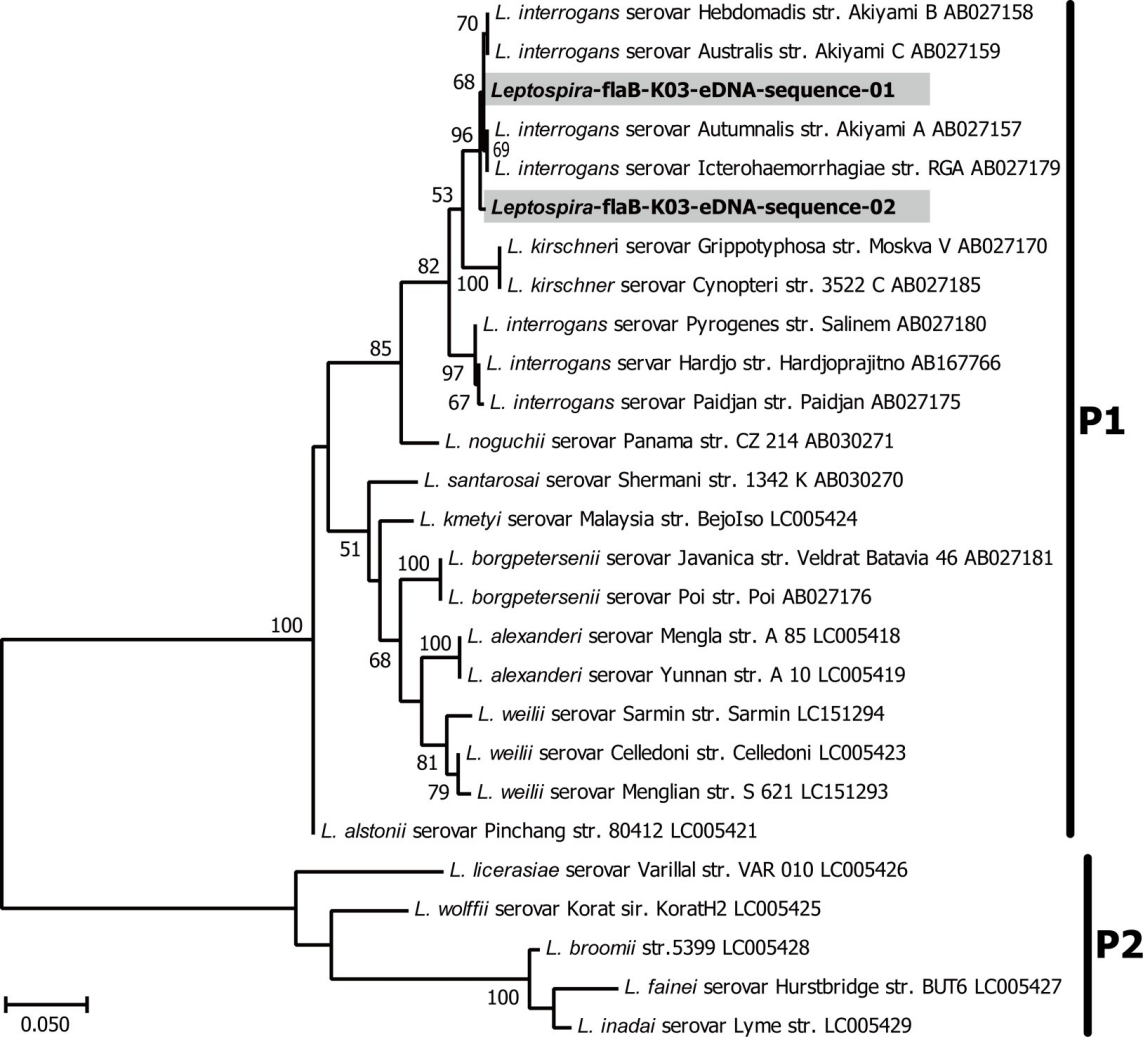
Molecular phylogenetic tree of leptospiral *flaB* genes. In total 432 nucleotide sites of *flaB* genes determined from environmental DNA analysis of the present study (shown in gray shading) were aligned and analyzed against known *flaB* sequences of representative *Leptospira* species. The GenBank accession numbers of the reference sequences were shown within sequence names. Maximum-likelihood phylogenetic analysis was performed with GTR + G + I model of nucleotide substitution. Numbers on the tree indicate support values for the nodes estimated from 100 bootstrap replications. K03 denotes the sample name collected from Kandy.

Next, to improve taxonomic resolution of the P1 subclade we targeted *flaB* gene [34, 35]. This less sensitive but conservative method yielded a PCR product from K03 sample and produced 3,696 and 2,708 of non-singleton, effective sequences from PCR replicate 1 and 2, respectively (for details, see Methods; DRA009845). These sequences comprised of two major ones with sequence counts of 1,904 (29.7%) and 4,500 (70.3%) and were denoted as *Leptospira*-flaB-K03-eDNA-sequence-01 and -02 in Fig 3, respectively. Phylogenetic analysis of these environmental *flaB* sequences with known *Leptospira flaB* sequences showed monophyletic clustering of environmental ones with those of *L*. *interrogans* (Fig 3).

### Environmental DNA detection of vertebrates correlated with *Leptospira*

To investigate the potential reservoir animals of *Leptospira* from the eDNA samples, we amplified partial fragments of the vertebrate mt12S rRNA gene and sequenced by the MiSeq using a Nano flowcell (maximum, 1 million reads). We obtained in total 330,573 sequence reads (4,132 ± 293 per a replicate) derived from PCR amplification of the 20 water samples (DRA009846). In total, 48,144 sequences remained after primary data processing, and other 282,429 reads were filtered due to an atypical short sequence length (< 204 bp; total 281,997 reads) or absence of primer sequences (total, 432 reads). From these de-noised sequences, we identified 33,090 non-singleton, effective sequences, and obtained in total 24,050 true-positive sequences (1,203 ± 214 per sample) after the exclusion of marine fish sequences (Fig 2; panels denoted as mammals, avians, frogs, and teleost fishes). Species annotation by a customized version of MiFish pipeline [50] with tetrapod data from the NCBI nucleotide database [42] yielded a total of 38 vertebrate species including 13 mammals, 2 avians, 1 frog, and 22 teleost fishes.

**Fig 4:**
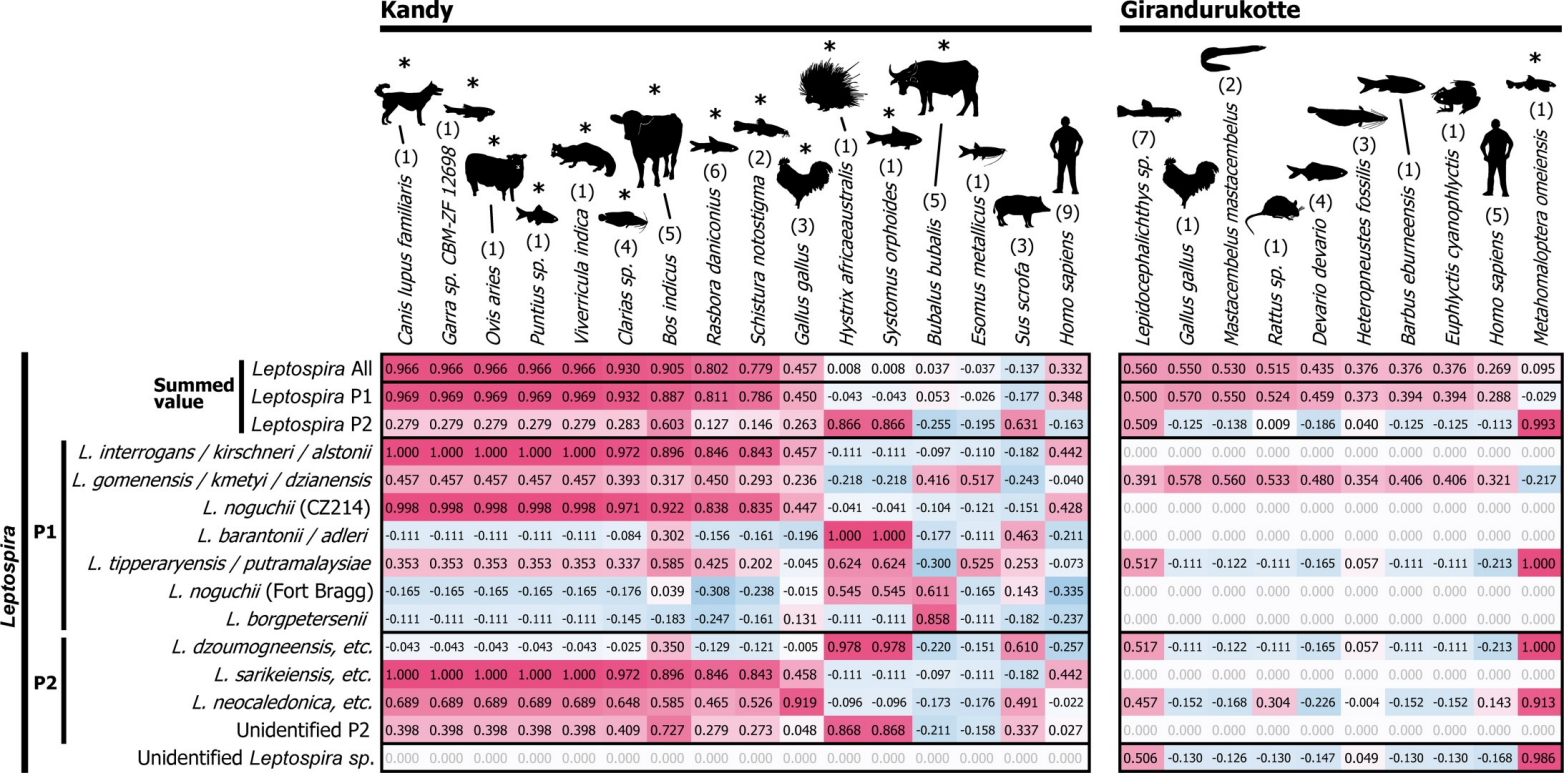
Correlation of *Leptospira* and vertebrate animals. Pearson’s correlation coefficients between detected read numbers of *Leptospira* (results from leptospiral 16S rRNA) and those of the vertebrates (results from mitochondrial 12S rRNA) were indicated in red (positive value) to blue (negative value) shading. The vertebrate species that showed positive correlation of *r* > 0.3 with at least one *Leptospira* OTU were shown. Asterisks indicate vertebrates that exhibited the significant correlation after Benjamini–Hochberg correction of false discovery rate at *q* < 0.01 in either row. P1 and P2 denote phylogenetic subclades of *Leptospira*. Numbers in the parenthesis above the scientific name of animals indicate number of times of appearance of the species in total of 10 water samples from the respective locations.

Among these vertebrates, we found that 13 and 1 species showed significant correlation with the eDNA detection of *Leptospira* in K and GK samples, respectively (Fig 4). These included animals such as dog (*Canis lupus familiaris*), sheep (*Ovis aries*), Indian civet (*Viverricula indica*), cattle (*Bos indicus*), chicken (*Gallus gallus*), porcupine (*Hystrix sp*.), water buffalo (*Bubalus bubalis*) (*r* > 0.632, *d*.*f*. = 8, *p* < 0.05; Benjamini–Hochberg [BH]-corrected false discovery rate [FDR] *q* < 0.01, *r* > 0.786 and *r* > 0.982 in K and GK, respectively) exhibiting significant correlation with at least one Operational Taxonomic Unit (OTU) of *Leptospira*. Boar (*Sus scrofa*), human (*Homo sapiens*), and rats (*Rattus sp*.) indicated positive but not significant correlations (*r* > 0.3) with some *Leptospira*. When focusing on tetrapod species detected more than two times (see parentheses denoted above the scientific names in Fig 4), cattle exhibited high correlation with several species of *Leptospira* (*e*.*g*., *r* = 0.896, *d*.*f*. = 8, *p* = 0.003 with *L*. *interrogans* group and *r* = 0.922, *d*.*f*. = 8, *p* = 0.001 with *L*. *noguchii* CZ214), whereas chicken and water buffalo showed high correlation with a particular OTU of *Leptospira* in Kandy (*r* = 0.919, *d*.*f*. = 8, *p* = 0.001 with *L*. *neocaledonica* group and *r* = 0.858, *d*.*f*. = 8, *p* = 0.006 with *L*. *borgpetersenii* group, respectively). In GK, on the other hand, there were no tetrapod animals that appeared repeatedly and exhibited high correlation with *Leptospira* detection.

### Environmental DNA analysis of bacterial correlated with *Leptospira* and vertebrates

From the multiplex metabarcoding sequences of universal bacterial 16S rRNA gene V4 region amplicons described in earlier section of the Results, we analyzed the environmental bacteria and candidate species associated with *Leptospira* in K and GK. From the 3,268,705 putative bacterial 16S rRNA gene V4 sequences, we identified 2,150,294 reliable ones that were repeatedly sequenced 10 times or more (approximately 200 counts per million [cpm] on average) in at least one replicate. A total of 456 bacterial species were obtained from 2,136,187 sequences (106,809 ± 5,647 sequences per sample; S2 Table). The remaining 14,107 (353 ± 42 sequences per sample) yielded no hits through Blast-based analysis against the GreenGenes database [43]. A non-metric multidimensional scaling plot of the standardized bacteriome of each sample (S3 Fig) indicated that the two sampling regions (K and GK) had distinct bacterial microbiota, which largely clustered according to the origin of samples.

**Fig 5:**
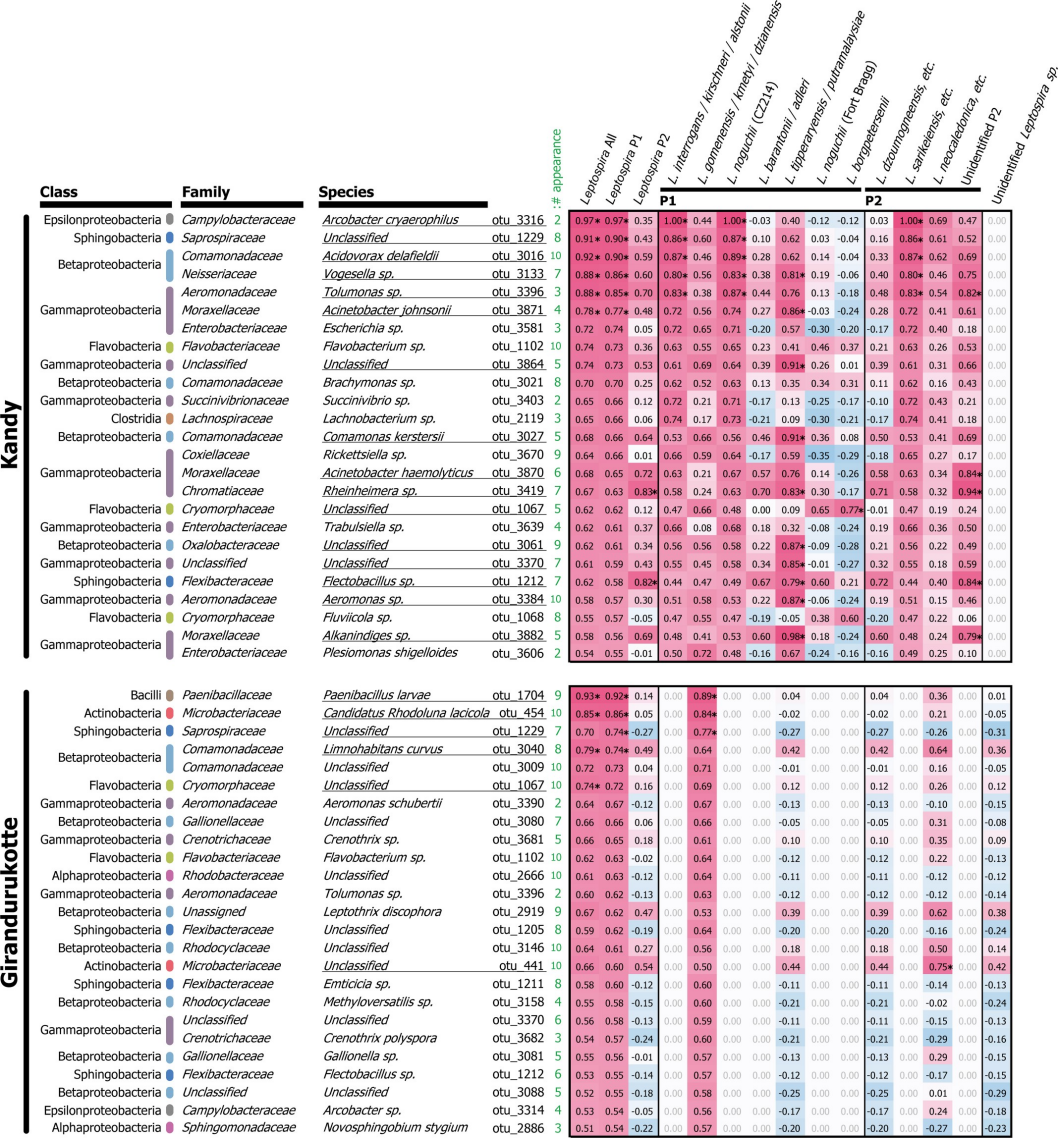
Correlation of *Leptospira* and other bacteria in environmental water. Pearson’s correlation coefficients between detected read numbers of *Leptospira* (results from leptospiral 16S rRNA) and those of bacteria (results from 16S rRNA V4 region) are indicated in red (positive value) to blue (negative value) shading. These bacteria are the top 25 species ordered by positive correlation values with summed counts of P1 *Leptospira* in respective regions. Asterisks indicate significant correlation after Benjamini–Hochberg correction of false discovery rate at *q* < 0.01. Underlines denote the bacteria which showed significant correlation with at least one of the columns of *Leptospira* OTUs or those of summed counts. P1 and P2 denote phylogenetic subclades of *Leptospira*. Numbers colored in green indicate number of times of appearance of the species in total of 10 water samples of respective regions.

Among these detected bacteria, 16 and 6 species exhibited significant correlation with the eDNA detection of pathogenic *Leptospira* in K and GK samples, respectively (*r* > 0.632 *d*.*f*. = 8, *p* < 0.05; BH-corrected FDR *q* < 0.01, *r* > 0.767 and *r* > 0.7395 in K and GK, respectively; indicated by underlines in Fig 5). Fig 5 shows top 25 detected bacterial species in each agro-ecological region, ordered by correlation between summed sequence counts of the P1 *Leptospira* (indicated in Fig 2). We found that *Arcobacter cryaerophilus* (OTU number 3316 in GreenGenes database) shows the highest correlation in K samples (*r* = 0.971, *d*.*f*. = 8, *p* < 0.001) and *Acinetobacter johnsonii* (OTU number 3871) show higher correlation (*r* = 0.767, *d*.*f*. = 8, *p* = 0.010) with P1 subclade than P2 subclade. *Acinetobacter haemolyticus* (OTU number 3870) indicated specifically high correlation with Unidentified P2 *Leptospira* (*r* = 0.839, *d*.*f*. = 8, *p* = 0.002), and *Aeromonas sp*. (OTU number 3384) also showed particularly high correlation with *L*. *tipperaryensis* group (*r* = 0.869, *d*.*f*. = 8, *p* = 0.001). In GK samples, on the other hand, there were only few bacteria species that exhibit significant correlation with pathogenic *Leptospira* (*e*.*g*., *Paenibacillus larvae*, OTU number 1704, *r* = 0.922, *d*.*f*. = 8, *p* < 0.001 and *Candidatus Rhodoluna lacicola*, OTU number 454, *r* = 0.860, *d*.*f*. = 8, *p* = 0.001).

We further addressed the true correlation of bacteria with *Leptospira* and vertebrate animals based on the partial correlation analysis and found that *A*. *cryaerophilus* (OTU number 3316 in Fig 5 and GreeGenes database) indicates a strong association with *Leptospira* (S4 Fig). In general, the bacteria that correlate with *Leptospira* (shown in Fig 5) also exhibit indirect correlation with animals that correlated with *Leptospira* (shown in Fig 4). Such cross-interfering, confounded correlations are commonly difficult to separate regarding noisy biological data and non-large sample sizes as in the present study [24, 49]. The partial correlations of vertebrate species with *Leptospira* P1 corrected by bacterial correlation remained higher in most of the vertebrates in K samples (yellow shading in an upper panel of the S4 Fig). On the other hand, the results did not show clear trends or any remarkable finding in GK samples with fewer bacterial and vertebrate OTUs correlated with *Leptospira* (a lower panel of the S4 Fig). Exceptionally, the partial correlation corrected by *A*. *cryaerophilus* exhibited lower values between most of the vertebrates and *Leptospira* (–0.32 to 0.38), indicating particular association of this bacterium with *Leptospira*. In addition, P1 *Leptospira* correlation with water buffalo became significant after correction by *A*. *cryaerophilus* (partial correlation *r* = 0.68, estimated *p* = 0.014), potentially indicating particular relationship among this bacterium, water buffalo, and *Leptospira*.

## Discussion

Most of the paddy lands in Sri Lanka are cultivated by either rain or irrigated water. It is a common practice in agricultural regions that potential pathogenic *Leptospira* reservoirs such as roaming livestock animals (buffalo and cattle) or domestic animals shared the irrigation water sources. These water streams are opened surface water channels which can be easily contaminated with surface pollutants including animal urine or feces, after precipitation (Fig 1B and 1C). Climatic factors are of primary importance in explaining the occurrence and diversity of human pathogens [51]. Sri Lanka is divided into different agro-ecological regions according to a particular combination of climate, soil and terrain conditions in order to design and evaluate agricultural research, land use and development strategies [14]. In this study, we analyzed two of these agro-ecological regions of Sri Lanka by eDNA metabarcoding to understand the eco-epidemiology of leptospirosis. The total species (bacterial and animal) diversity is greater in Kandy (wet region mid-country region 2) than in Girandurukotte (intermediate region low-country region 2) (Fig 2). Therefore, our results showed that the differences in agro-ecological regions can impact on the diversity of *Leptospira* species, correlated environmental bacteria and reservoir animal species. Since the population density of cattle and buffalo is similar in both Kandy and Badulla districts, our results suggested that the greater number of sequence-reads detected in each sample is a consequence of different agro-ecological regions.

The diagnosis of the great majority of the reported cases in Sri Lanka is not laboratory confirmed, thus they can be categorized as clinical suspected cases of leptospirosis. Based on this report system, the annual national leptospirosis incidence is 5.4 per 100,000 population [51–53]. Clinical suspected leptospirosis cases have been reported in the sampling areas [54, 55]. In 2018, 124 cases in Kandy and 189 in Badulla, of which, 12 and 21 were reported on December for Kandy and Badulla (GK), respectively. The higher number of human leptospirosis cases in the intermediate region than in the wet region is not correlated with our environmental metabarcoding findings, but we can hypothesized that the higher incidence of leptospirosis-like acute febrile diseases (such as hantavirus infection) in GK than in Kandy is a factor that led to an overestimation of clinical suspected cases of leptospirosis in this particular area [56–57]. Our study emphasizes the need to have an accurate laboratory-based diagnostic system in Sri Lanka to properly control both infectious diseases. This study showed, for the first time, the great *Leptospira* species diversity in Kandy. The species reported to be etiological agents of human leptospirosis in Sri Lanka are *L*. *interrogans*, *L*. *kirschneri*, *L. borgpetersenii*, *L*. *santarosai*, and *L*. *weilii* belonging to P1 subclade [22]. However, *L*. *noguchii* belonging to the P1-virulent group was also detected in Kandy suggesting that the etiological agents of leptospirosis in Sri Lanka might be underestimated. Interestingly, *Leptospira* species detected in Girandurukotte, belong to the P1 subclade-low virulent group and P2 subclade and we could not detect any correlation with tetrapod animals which support the idea that these *Leptospira* species might not need a mammal reservoir in their biology [2]. We detected clinically important species only in Kandy and by 16S rRNA sequencing they grouped together as *L*. *interrogans*/*L*. *kirschneri* /*L. alstonii* (Fig 2). The taxonomy of *Leptospira* was recently revisited by the addition of 30 novel species and it was shown that the 16S rRNA gene discriminatory power is not sufficient to distinguish *Leptospira* species [1]. Vincent *et al*. reported that the *ppk* gene, encoding a polyphosphate kinase, effectively made it possible to recover the monophyly of the P1 subclade, thus, *ppK* warrant further investigation as a candidate for eDNA barcoding [1]. In this study, to discriminate the species within the *L*. *interrogans/ L*. *kirschneri/ L*. *alstonii* group we targeted the flagellar gene *flaB*. Although *flaB* eDNA detection was not as sensitive as 16S rRNA, eDNA sequences were detected in K03 which belonged to *L*. *interrogans*, an etiological agent of leptospirosis in Sri Lanka (Fig 3). The analysis of 16S rRNA gene showed that our method detected environmental pathogenic *Leptospira* species and could differentiate P1 and P2 subclades in a wild biome of an endemic area of Japan and in agricultural areas of Sri Lanka [24]. However, environmental detection of *lipL32* gene might be greatly influenced by the presence of other bacteria and/or environmental factors since *lipL32* detection showed low sensitivity and specificity in this study (S2 Fig). Non-leptospiral *lipL32* sequences were more abundant in the environment than the leptospiral ones, suggesting that *lipL32* orthologs in environmental ecosystems may be greater than previously estimated [24]. Thus, although *lipL32* gene is a good marker to detect pathogenic *Leptospira* when dealing with clinical samples, the wide distribution of *lipL32* orthologs in the environment should be considered when dealing with environmental samples and highly sensitive methods such as next-generation sequencing [24]. Our results suggested that 16S rRNA is the best target to be detected in a wide range of environments.

Sequencing of mt 12S rRNA gene allowed us to detect local animals (Fig 2), however, discrimination between closely related animals was difficult (*i.e.*, sheep vs. goat). Because sheep is not an endemic, widespread mammal in Kandy, the mt DNA sequence detected as sheep might be derived from goat (*Capra aegagrus*). The diversity of animal species detected in K samples was greater than in GK samples. Kandy also showed more animal species associated with pathogenic bacterial species than Girandurukotte (Fig 4). Interestingly, when analyzing the correlation of animals with pathogenic *Leptospira*, known *Leptospira* reservoirs in wild biome such as boar (*Sus scrofa*) [58] and urban areas such as rats (*Rattus sp*.) [59] did not show any correlation in this study. On the other hand, draught animals used in agriculture such as cattle (*Bos indicus*) and water buffalo (*Bubalus bubalis*) showed a high correlation with pathogenic *Leptospira*. Interestingly, while cattle association was high with several *Leptospira* species, water buffalo association was high only with *L*. *borgpetersenii* suggesting a more restricted host preference for this species in Sri Lanka (Fig 4). *L*. *borgpetersenii* have been associated with water buffalo in several reports [60, 61]. Since large mammals that excrete much more urine than rats shed significantly more *Leptospira* per day, our results suggested that large mammals are the main contaminating source of irrigation water in Kandy. However, the association of large mammals with *Leptospira* barcodes should be interpreted with caution because large mammals wading in surface waters (for drinking or for draught activities) also resuspend soil and sediment particles, this association could be indirect reflecting the resuspension of local bacteria by chance and not an association with reservoir animals.

A study, using the eDNA metabarcoding method did not detect human eDNA in two rivers of endemic areas of Japan [24] In this study, human eDNA was detected in 9 samples of Kandy (358 sequence-reads) and 5 samples of Girandurukotte (131 sequence-reads), which showed the high interaction of human with irrigation water (where people get bath and wash their clothes) and the less dilution effect of irrigation water when comparing with rivers, both effects might increase the risk of infection in the paddy lands of Sri Lanka. The difference observed in human eDNA detection in both areas might reflect the difference in human population density which is higher in Kandy and not only differences of agro-ecological regions [30]. We also detected marine fish sequences, although these sequences were excluded from the present analysis as apparent noise data. Some detected species, however, appeared to be derived from canned foods consumed in the sampling regions (*i*.*e*., *Scomberomorus commerson* and *Thunnus albacares*). Thus, the detection of vertebrates by the eDNA metabarcoding method is also a useful tool for evaluating human activities and monitoring potential environmental pollution.

The analysis of universal 16S rRNA bacterial region showed distinct bacterial microbiota in the two sampling regions (K and GK) (S3 Fig). Several emerging pathogenic bacterial species (*i.e.*, *Arcobacter cryaerophylus*, *Aeromonas* sp.), some of them not yet isolated in Sri Lanka, were detected mainly in Kandy (Fig 5). *A*. *cryaerophylus* showed the highest correlation with the *L*. *interrogans/kirschneri/alstonii* group. *Arcobacter* has commonly been isolated from feces and rectal swabs of healthy cattle and other animals [62]. However, *Arcobacter* has becoming an emerging threat since it has also been associated with abortion and reproductive problems in various production animals and from cases of diarrhea and septicaemia in humans [63]. *A*. *cryaerophylus* can adapt and survive readily in environmental water, thus, water has a significant role in the transmission of *Arcobacter* species both to animals and humans. Therefore, the water contamination of irrigation water of Kandy might be of significance to human health [64]. The high correlation of fecal-derived bacterial species (*A*. *cryaerophylus*, *Escherichia* sp.) with urine-derived bacterial species (*Leptospira* spp.) in Kandy but not in Girandurukotte, suggested that the contamination of irrigation water with these bacteria is dependent on the climate and soil characteristics of each agro-ecological region, supporting that floods and rainfall are important drivers that increase the risk of infection on islands and in Asia [7, 65]. Interestingly, the correlation of fecal-derived bacterial species is greater with *Leptospira* P1 than *Leptospira* P2. Among *Leptospira* P1, clinically important species (*L*. *interrogans*, *L*. *kirschneri*) showed the highest correlation with *A*. *cryaerophylus*. This high correlation might be due to the simultaneously fecal and urine contamination and/or the capability of both genera to form biofilms [66, 67].

Other potential pathogens such as *Acinetobacter johnsonii* and *A*. *haemolyticus*, showed also high correlation with *Leptospira* species in Kandy (Fig 5). *Acinetobacter* spp. have the ability to occupy several ecological niches including environment, animals and human [68] The most common species to cause infection is *A*. *baumannii*, additional species such as *A*. *haemolyticus* and *A*. *johnsonii* have occasionally been reported as pathogens [69]. Environmental *Acinetobacter* strains often harbor antibiotic resistance genes that might transform into clinically relevant strains [69].

In conclusion, this study shows the usefulness of the eDNA metabarcoding method to compare the microbiome of different regions and, particularly, for the environmental surveillance of clinical important leptospiral species that are difficult to culture, and which isolation is time consuming. Furthermore, the correlation of pathogenic *Leptospira* with potential animal reservoirs in Kandy confirmed that the wet region facilitates leptospirosis transmission. The eDNA analysis also showed unexpected leptospiral correlation with other pathogenic bacterial species that have not been reported in Sri Lanka but might have importance for public health policies. Our data provide a platform to understand the eco-epidemiology of leptospirosis in Sri Lanka, where the diverse climate and large number of reservoir animals in each region made it difficult to implement disease control activities.

## Supporting information

S1 Fig Molecular phylogenetic tree of leptospiral 16S rRNA genes. In total 280 nucleotide sites of partial leptospiral 16S rRNA genes determined from environmental DNA analysis of the present study (shown in blue shading) were aligned and analyzed with known reference 16S rRNA sequences of representative species of *Leptospira* (indicated by pink and orange letters). The GenBank accession numbers of the reference sequences were shown within sequence names. Maximum-likelihood phylogenetic analysis was performed with GTR + G + I model of nucleotide substitution. Numbers on the tree indicate support values for the nodes estimated from 100 bootstrap replications. K and GK indicate sampling locations Kandy and Girandurukotte, respectively; K-01−K-10 and GK-01−GK-10 indicate sample names. The orange lettered species indicate that their species annotation were corrected based on this phylogenetic analysis in the present study.
(PDF)

S2 Fig Environmental detection of Leptospira lipL32 gene. Number of sequence reads detected in each sample are shown with colored matrices in red shading for *Leptospira*, blue shading for other putative bacteria, and gray shading for the sequences with no database hit in BLAST-based analysis, respectively. Sequence counts from two PCR replicates were summed up for each sample. K and GK indicate sampling locations Kandy and Girandurukotte, respectively. K01−K10 and GK01−GK10 denote sample names.
(PDF)

S3 Fig Bacteriome profiles of water samples of Kandy and Girandurukotte. Ordinations of two-dimensional nonmetric multidimensional scaling of the standardized profiles of bacteriomes were estimated from the 16S rRNA gene V4 region data for each sample. K and GK indicate sampling locations Kandy and Girandurukotte, respectively; K-01−K-10 and GK-01−GK-10 indicate sample names. Horizontal and vertical axes correspond to the estimated two-dimensional coordinate 1 and 2 where ranked differences in similarity scores on the basis of Pearson's correlation coefficients (*r*) were preserved. A normalized stress value of this plot was 0.163, and the determination factor *R^2^* values of coordinate 1 and 2 were 0.805 and 0.243, respectively.
(PDF)

S4 Fig Correlation between bacteria and vertebrate animals and partial correlation between vertebrates and P1 *Leptospira* corrected for the bacteria. Pearson’s correlation coefficients between detected read numbers of bacteria (results from bacterial 16S rRNA gene V4 region) and those of the vertebrates (results from mitochondrial 12S rRNA) were indicated in red (positive value) to blue (negative value) shading. Numbers in green letters next to bacterial OTU numbers of the GreenGenes database indicate number of times of appearance of the OTUs in total of 10 water samples. Partial correlation coefficients between summed read numbers of P1 *Leptospira* (shown in Fig 2) and read numbers of vertebrates were indicated after correction by the read numbers of bacterial 16S rRNA gene V4. Yellow to green shading denote positive to negative values of partial correlation, respectively.
(PDF)

S1 Table Concentration and quality of the extracted DNA. (XLSX)

S2 Table Sequence counts and taxonomic profiling of bacterial species detected from water samples. (XLSX)
