# Supplementary figures and images for: Understanding leptospirosis eco-epidemiology by environmental DNA metabarcoding of irrigation water from two agro-ecological regions of Sri Lanka
Source: PLoS Negl Trop Dis. 2020 Jul 23;14(7):e0008437. doi: 10.1371/journal.pntd.0008437 (PMC7377381; doi:10.1371/journal.pntd.0008437)

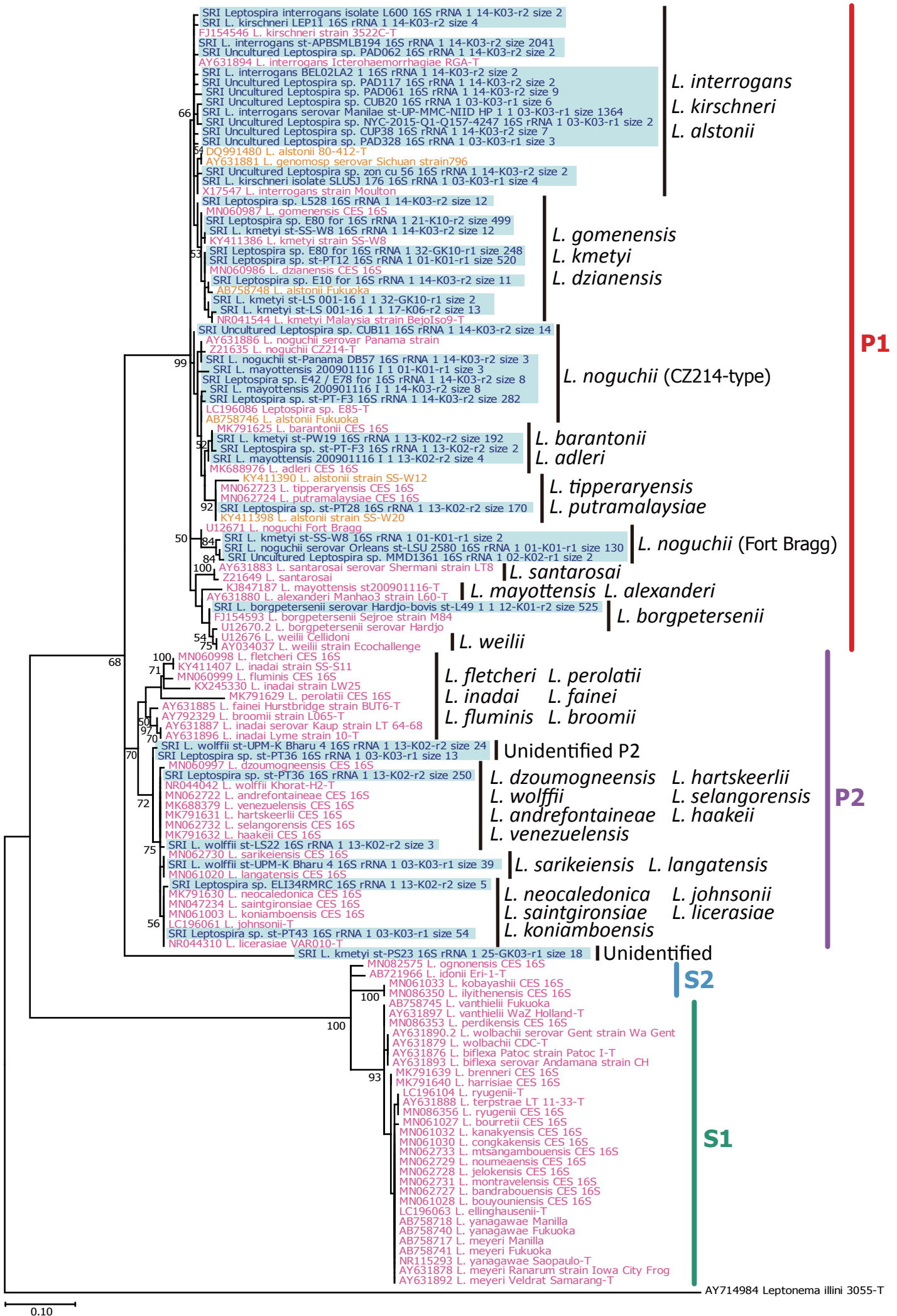

Supplementary Fig. S1

Supplement: S1 Fig — In total 280 nucleotide sites of partial leptospiral 16S rRNA genes determined from environmental DNA analysis of the present study (shown in blue shading) were aligned and analyzed with known reference 16S rRNA sequences of representative species of Leptospira (indicated by pink and orange letters). The GenBank accession numbers of the reference sequences were shown within sequence names. Maximum-likelihood phylogenetic analysis was performed with GTR + G + I model of nucleotide substitution. Numbers on the tree indicate support values for the nodes estimated from 100 bootstrap replications. K and GK indicate sampling locations Kandy and Girandurukotte, respectively; K-01−K-10 and GK-01−GK-10 indicate sample names. The orange lettered species indicate that their species annotation were corrected based on this phylogenetic analysis in the present study. (PDF) [file pntd.0008437.s001.pdf]

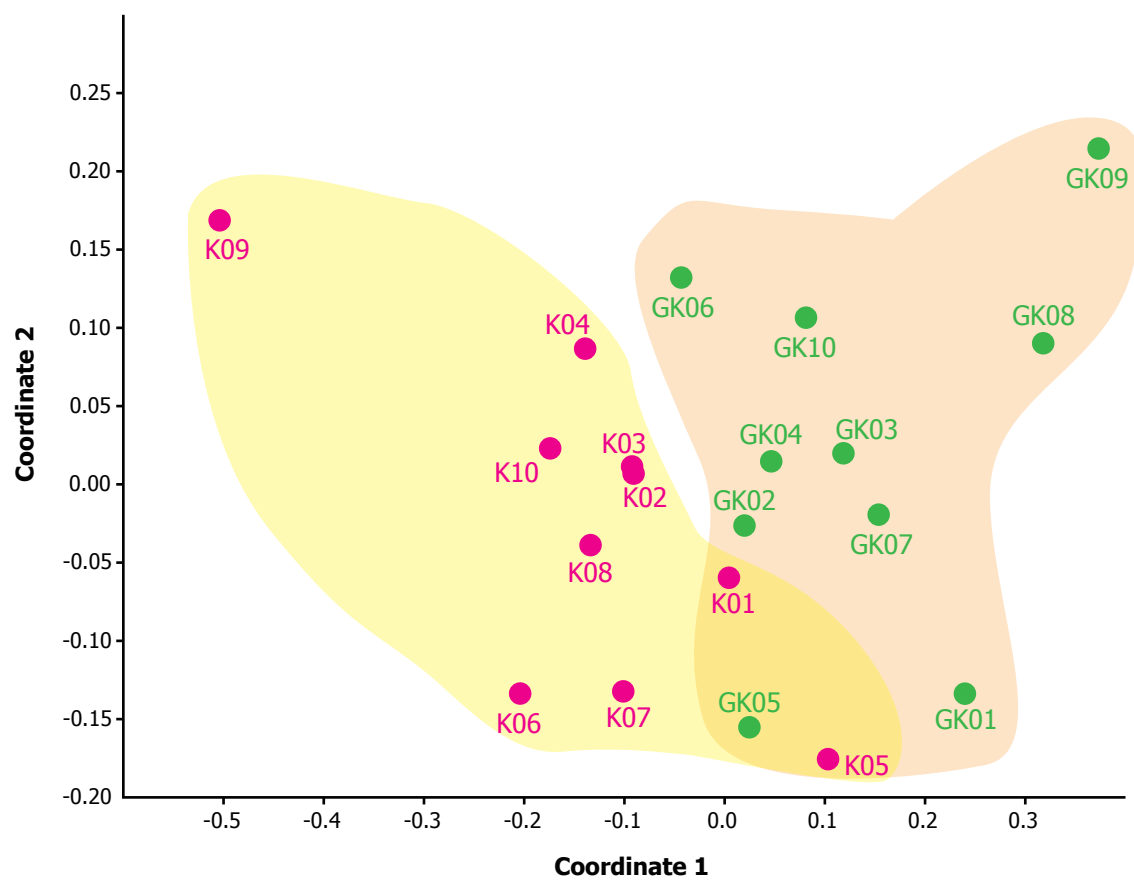

Supplementary Fig. S3

Supplement: S3 Fig — Ordinations of two-dimensional nonmetric multidimensional scaling of the standardized profiles of bacteriomes were estimated from the 16S rRNA gene V4 region data for each sample. K and GK indicate sampling locations Kandy and Girandurukotte, respectively; K-01−K-10 and GK-01−GK-10 indicate sample names. Horizontal and vertical axes correspond to the estimated two-dimensional coordinate 1 and 2 where ranked differences in similarity scores on the basis of Pearson's correlation coefficients (r) were preserved. A normalized stress value of this plot was 0.163, and the determination factor R2 values of coordinate 1 and 2 were 0.805 and 0.243, respectively. (PDF) [file pntd.0008437.s003.pdf]
