# Supplementary material for: Understanding leptospirosis eco-epidemiology by environmental DNA metabarcoding of irrigation water from two agro-ecological regions of Sri Lanka
Source: PLoS Negl Trop Dis. 2020 Jul 23;14(7):e0008437. doi: 10.1371/journal.pntd.0008437 (PMC7377381; doi:10.1371/journal.pntd.0008437)
Supplement: S2 Fig — Number of sequence reads detected in each sample are shown with colored matrices in red shading for Leptospira, blue shading for other putative bacteria, and gray shading for the sequences with no database hit in BLAST-based analysis, respectively. Sequence counts from two PCR replicates were summed up for each sample. K and GK indicate sampling locations Kandy and Girandurukotte, respectively. K01−K10 and GK01−GK10 denote sample names. (PDF) [file pntd.0008437.s002.pdf]

		Leptospira			Other putative bacteria											
		L. interrogans	*L. kmetyi*	*Uncultured Leptospira sp.*	*Acidovorax sp.*	*Aurantimicrobium sp.*	*Chryseobacterium balustinum*	*Chryseobacterium sp.*	*Colletotrichum graminicola*	*Herbaspirillum robiniae*	*Herbaspirillum seropedicae*	*Hydrogenophaga sp.*	*Melaminivora sp.*	*Ottowia sp.*	*Unknown Anopheles gambiae related*	*No Blast hit*
**Kandy**	K01	0	0	0	0	0	0	0	0	0	0	0	0	29	0	2276
	K02	0	733	0	6	0	0	0	0	0	0	0	0	2	0	1793
	K03	4508	0	9	0	0	0	0	33	0	0	0	35	0	0	825
	K04	0	0	0	0	0	0	2	0	0	0	0	0	0	0	553
	K05	0	0	0	18	0	0	0	0	0	0	0	0	0	0	398
	K06	0	0	0	0	0	0	0	0	0	0	0	0	0	0	2599
	K07	0	0	0	5	0	0	0	322	0	0	0	0	0	2	790
	K08	0	0	0	0	0	0	0	0	0	0	0	0	0	0	181
	K09	0	0	0	0	0	0	0	0	0	0	0	0	0	0	233
	K10	0	0	359	106	0	3	2	2	0	0	0	0	8	0	2037
	K-Nega	0	0	0	0	0	0	0	0	0	0	0	0	0	0	0
**Giradurukotte**	GK01	0	0	0	0	0	0	0	0	0	0	0	0	0	0	684
	GK02	0	0	0	0	0	0	0	0	0	0	0	0	0	0	453
	GK03	0	0	0	0	0	0	0	9	0	0	6	0	0	0	6072
	GK04	0	0	0	0	7	0	0	0	67	2	0	0	0	0	5495
	GK05	0	0	0	0	0	0	0	0	0	0	0	0	0	0	1340
	GK06	0	0	0	0	0	0	0	0	0	0	0	0	0	0	67
	GK07	0	0	0	0	0	0	0	0	0	0	0	0	0	0	137
	GK08	0	0	0	0	0	0	0	0	0	0	0	0	0	0	15
	GK09	0	0	0	0	0	0	0	0	0	0	0	0	0	0	118
	GK10	0	0	0	0	0	0	0	0	0	0	0	0	0	0	179
	GK-Nega	0	0	0	0	0	0	0	0	0	0	0	0	0	0	2

Supplementary Fig. S2
