# Supplementary material for: Understanding leptospirosis eco-epidemiology by environmental DNA metabarcoding of irrigation water from two agro-ecological regions of Sri Lanka
Source: PLoS Negl Trop Dis. 2020 Jul 23;14(7):e0008437. doi: 10.1371/journal.pntd.0008437 (PMC7377381; doi:10.1371/journal.pntd.0008437)
Supplement: S4 Fig — Pearson’s correlation coefficients between detected read numbers of bacteria (results from bacterial 16S rRNA gene V4 region) and those of the vertebrates (results from mitochondrial 12S rRNA) were indicated in red (positive value) to blue (negative value) shading. Numbers in green letters next to bacterial OTU numbers of the GreenGenes database indicate number of times of appearance of the OTUs in total of 10 water samples. Partial correlation coefficients between summed read numbers of P1 Leptospira (shown in Fig 2) and read numbers of vertebrates were indicated after correction by the read numbers of bacterial 16S rRNA gene V4. Yellow to green shading denote positive to negative values of partial correlation, respectively. (PDF) [file pntd.0008437.s004.pdf]

Kandy

Pearson' s correlation coefficient *r* between bacteria and vertebrates (colour scale: 1.0 to −1.0)

Class	Family	Species	OTU	# appearance	*Canis lupus familiaris*	*Garra sp CBM-ZF 1269*	*Ovis aries*	*Puntius sp.*	*Viverricula indica*	*Clarias sp.*	*Bos indicus*	*Rasbora daniconius*	*Schistura notostigma*	*Gallus gallus*	*Hystrix africaeaustralis*	*Systomus orphoides*	*Bubalus bubalis*	*Esomus metallicus*	*Sus scrofa*	*Homo sapiens*
Epsilonproteobacteria	*Campylobacteraceae*	*Arcobacter cryaerophilus*	otu_3316	2	1.00	1.00	1.00	1.00	1.00	0.97	0.92	0.84	0.83	0.44	-0.03	-0.03	-0.11	-0.12	-0.15	0.43
Sphingobacteria	*Saprospiraceae*	*Unclassified*	otu_1229	8	0.86	0.86	0.86	0.86	0.86	0.83	0.81	0.77	0.69	0.42	0.10	0.10	-0.07	0.16	0.00	0.26
Betaproteobacteria	*Comamonadaceae*	*Acidovorax delafieldii*	otu_3016	10	0.87	0.87	0.87	0.87	0.87	0.86	0.94	0.73	0.72	0.41	0.28	0.28	-0.05	-0.05	0.06	0.30
Betaproteobacteria	*Neisseriaceae*	*Vogesella sp.*	otu_3133	7	0.80	0.80	0.80	0.80	0.80	0.80	0.91	0.76	0.66	0.22	0.38	0.38	-0.08	0.18	0.01	0.27
Gammaproteobacteria	*Aeromonadaceae*	*Tolumonas sp.*	otu_3396	3	0.83	0.83	0.83	0.83	0.83	0.81	0.96	0.71	0.65	0.27	0.44	0.44	-0.22	0.01	0.09	0.25
Gammaproteobacteria	*Moraxellaceae*	*Acinetobacter johnsonii*	otu_3871	4	0.72	0.72	0.72	0.72	0.72	0.74	0.78	0.82	0.65	0.15	0.27	0.27	-0.19	0.43	0.01	0.29
Gammaproteobacteria	*Enterobacteriaceae*	*Escherichia sp.*	otu_3581	3	0.72	0.72	0.72	0.72	0.72	0.67	0.59	0.80	0.54	0.19	-0.20	-0.20	-0.25	0.53	-0.21	0.30
Flavobacteria	*Flavobacteriaceae*	*Flavobacterium sp.*	otu_1102	10	0.63	0.63	0.63	0.63	0.63	0.61	0.75	0.50	0.50	0.19	0.23	0.23	0.31	-0.12	-0.18	0.28
Gammaproteobacteria	*Unclassified*	*Unclassified*	otu_3864	5	0.61	0.61	0.61	0.61	0.61	0.59	0.72	0.64	0.46	0.13	0.39	0.39	-0.05	0.47	0.06	0.08
Betaproteobacteria	*Comamonadaceae*	*Brachymonas sp.*	otu_3021	8	0.62	0.62	0.62	0.62	0.62	0.60	0.57	0.55	0.51	0.05	0.13	0.13	0.26	-0.09	-0.35	0.38
Gammaproteobacteria	*Succinivibrionaceae*	*Succinivibrio sp.*	otu_3403	2	0.72	0.72	0.72	0.72	0.72	0.68	0.61	0.64	0.56	0.23	-0.17	-0.17	-0.20	-0.17	-0.27	0.51
Clostridia	*Lachnospiraceae*	*Lachnobacterium sp.*	otu_2119	3	0.74	0.74	0.74	0.74	0.74	0.68	0.71	0.64	0.56	0.19	-0.21	-0.21	-0.26	-0.21	-0.34	0.56
Betaproteobacteria	*Comamonadaceae*	*Comamonas kerstersii*	otu_3027	5	0.53	0.53	0.53	0.53	0.53	0.49	0.66	0.49	0.32	0.29	0.46	0.46	-0.05	0.44	0.29	-0.14
Gammaproteobacteria	*Coxiellaceae*	*Rickettsiella sp.*	otu_3670	9	0.65	0.65	0.65	0.65	0.65	0.61	0.58	0.77	0.51	0.03	-0.17	-0.17	-0.32	0.58	-0.28	0.28
Gammaproteobacteria	*Moraxellaceae*	*Acinetobacter haemolyticus*	otu_3870	6	0.63	0.63	0.63	0.63	0.63	0.62	0.90	0.57	0.46	0.06	0.57	0.57	-0.32	0.02	0.10	0.26
Gammaproteobacteria	*Chromatiaceae*	*Rheinheimera sp.*	otu_3419	7	0.58	0.58	0.58	0.58	0.58	0.58	0.82	0.49	0.44	0.08	0.70	0.70	-0.22	0.02	0.19	0.12
Flavobacteria	*Cryomorphaceae*	*Unclassified*	otu_1067	5	0.47	0.47	0.47	0.47	0.47	0.41	0.40	0.19	0.30	0.30	0.00	0.00	0.62	-0.23	-0.24	-0.01
Gammaproteobacteria	*Enterobacteriaceae*	*Trabulsiella sp.*	otu_3639	4	0.66	0.66	0.66	0.66	0.66	0.81	0.72	0.81	0.90	0.11	0.18	0.18	0.10	-0.24	-0.15	0.76
Betaproteobacteria	*Oxalobacteraceae*	*Unclassified*	otu_3061	9	0.56	0.56	0.56	0.56	0.56	0.56	0.65	0.72	0.48	-0.03	0.22	0.22	-0.27	0.62	-0.06	0.25
Gammaproteobacteria	*Unclassified*	*Unclassified*	otu_3370	7	0.55	0.55	0.55	0.55	0.55	0.58	0.69	0.75	0.51	-0.08	0.34	0.34	-0.22	0.47	-0.06	0.39
Sphingobacteria	*Flexibacteraceae*	*Flectobacillus sp.*	otu_1212	7	0.44	0.44	0.44	0.44	0.44	0.40	0.66	0.29	0.22	0.33	0.67	0.67	0.05	0.10	0.42	-0.21
Gammaproteobacteria	*Aeromonadaceae*	*Aeromonas sp.*	otu_3384	10	0.51	0.51	0.51	0.51	0.51	0.52	0.59	0.71	0.45	-0.08	0.22	0.22	-0.22	0.66	-0.08	0.25
Flavobacteria	*Cryomorphaceae*	*Fluviicola sp.*	otu_1068	8	0.47	0.47	0.47	0.47	0.47	0.50	0.30	0.34	0.50	0.29	-0.19	-0.19	0.65	-0.22	-0.31	0.26
Gammaproteobacteria	*Moraxellaceae*	*Alkanindiges sp.*	otu_3882	5	0.48	0.48	0.48	0.48	0.48	0.48	0.69	0.56	0.36	0.00	0.60	0.60	-0.28	0.43	0.20	0.07
Gammaproteobacteria	*Enterobacteriaceae*	*Plesiomonas shigelloides*	otu_3606	2	0.49	0.49	0.49	0.49	0.49	0.45	0.40	0.65	0.36	0.10	-0.16	-0.16	-0.21	0.81	-0.11	0.08

Partial correlation between P1 *Leptospira* and vertebrates corrected for bacteria (colour scale: 1.0 to −1.0)

OTU	*Canis lupus familiaris*	*Garra sp CBM-ZF 12698*	*Ovis aries*	*Puntius sp.*	*Viverricula indica*	*Clarias sp.*	*Bos indicus*	*Rasbora daniconius*	*Schistura notostigma*	*Gallus gallus*	*Hystrix africaeaustralis*	*Systomus orphoides*	*Bubalus bubalis*	*Esomus metallicus*	*Sus scrofa*	*Homo sapiens*
otu_3316	0.04	0.04	0.04	0.04	0.04	-0.20	-0.12	-0.03	-0.19	0.08	-0.04	-0.04	0.68	0.38	-0.15	-0.32
otu_1229	0.88	0.88	0.88	0.88	0.88	0.76	0.62	0.42	0.53	0.19	-0.31	-0.31	0.26	-0.39	-0.41	0.26
otu_3016	0.87	0.87	0.87	0.87	0.87	0.71	0.27	0.52	0.46	0.21	-0.71	-0.71	0.23	0.05	-0.53	0.19
otu_3133	0.92	0.92	0.92	0.92	0.92	0.80	0.48	0.47	0.57	0.52	-0.79	-0.79	0.24	-0.36	-0.38	0.24
otu_3396	0.90	0.90	0.90	0.90	0.90	0.78	0.46	0.56	0.58	0.43	-0.89	-0.89	0.47	-0.06	-0.49	0.27
otu_3871	0.94	0.94	0.94	0.94	0.94	0.85	0.72	0.50	0.59	0.53	-0.40	-0.40	0.31	-0.62	-0.29	0.21
otu_3581	0.93	0.93	0.93	0.93	0.93	0.87	0.83	0.55	0.68	0.47	0.16	0.16	0.36	-0.73	-0.03	0.20
otu_1102	0.96	0.96	0.96	0.96	0.96	0.90	0.75	0.76	0.71	0.47	-0.32	-0.32	-0.27	0.09	-0.07	0.22
otu_3864	0.97	0.97	0.97	0.97	0.97	0.91	0.76	0.65	0.74	0.52	-0.51	-0.51	0.13	-0.60	-0.33	0.42
otu_3021	0.96	0.96	0.96	0.96	0.96	0.89	0.83	0.71	0.70	0.58	-0.19	-0.19	-0.19	0.05	0.10	0.13
otu_3403	0.95	0.95	0.95	0.95	0.95	0.88	0.82	0.67	0.67	0.41	0.09	0.09	0.25	0.11	0.00	0.01
otu_2119	0.95	0.95	0.95	0.95	0.95	0.88	0.79	0.67	0.67	0.44	0.13	0.13	0.31	0.15	0.06	-0.03
otu_3027	0.97	0.97	0.97	0.97	0.97	0.93	0.80	0.74	0.81	0.36	-0.52	-0.52	0.11	-0.47	-0.51	0.59
otu_3670	0.95	0.95	0.95	0.95	0.95	0.89	0.82	0.63	0.69	0.57	0.09	0.09	0.36	-0.66	0.01	0.22
otu_3870	0.95	0.95	0.95	0.95	0.95	0.89	0.91	0.70	0.72	0.54	-0.66	-0.66	0.36	-0.05	-0.32	0.24
otu_3419	0.95	0.95	0.95	0.95	0.95	0.89	0.83	0.74	0.73	0.52	-0.88	-0.88	0.25	-0.05	-0.39	0.35
otu_1067	0.98	0.98	0.98	0.98	0.98	0.95	0.89	0.90	0.80	0.35	-0.06	-0.06	-0.53	0.15	-0.04	0.45
otu_3639	0.95	0.95	0.95	0.95	0.95	0.94	0.81	0.68	0.67	0.49	-0.20	-0.20	-0.01	0.15	-0.11	-0.23
otu_3061	0.96	0.96	0.96	0.96	0.96	0.90	0.81	0.67	0.71	0.60	-0.24	-0.24	0.29	-0.66	-0.18	0.26
otu_3370	0.96	0.96	0.96	0.96	0.96	0.90	0.82	0.69	0.70	0.62	-0.32	-0.32	0.23	-0.43	-0.18	0.15
otu_1212	0.98	0.98	0.98	0.98	0.98	0.94	0.82	0.83	0.83	0.34	-0.71	-0.71	0.03	-0.10	-0.56	0.59
otu_3384	0.96	0.96	0.96	0.96	0.96	0.91	0.83	0.70	0.72	0.61	-0.21	-0.21	0.22	-0.67	-0.16	0.26
otu_1068	0.97	0.97	0.97	0.97	0.97	0.91	0.91	0.80	0.70	0.36	0.08	0.08	-0.51	0.12	0.00	0.25
otu_3882	0.96	0.96	0.96	0.96	0.96	0.91	0.84	0.73	0.76	0.54	-0.57	-0.57	0.26	-0.35	-0.35	0.37
otu_3606	0.96	0.96	0.96	0.96	0.96	0.92	0.87	0.71	0.76	0.48	0.06	0.06	0.21	-0.96	-0.14	0.37

Giradurukotte

Class	Family	Species	OTU	# appearance	*Lepidocephalichthys sp.*	*Gallus gallus*	*Mastacembelus mastacembelus*	*Rattus sp.*	*Devario devario*	*Heteropneustes fossilis*	*Barbus eburneensis*	*Euphlyctis cyanophlyctis*	*Homo sapiens*	*Metahomaloptera omeiensis*
Bacilli	*Paenibacillaceae*	*Paenibacillus larvae*	otu_1704	9	0.40	0.40	0.38	0.78	0.30	0.13	0.14	0.14	0.54	0.04
Actinobacteria	*Microbacteriaceae*	*Candidatus Rhodoluna lacicola*	otu_454	10	0.57	0.75	0.73	0.56	0.65	-0.14	-0.12	-0.12	0.37	-0.02
Sphingobacteria	*Saprospiraceae*	*Unclassified*	otu_1229	7	0.45	0.78	0.76	-0.02	0.75	0.34	0.41	0.41	-0.08	-0.27
Betaproteobacteria	*Comamonadaceae*	*Limnohabitans curvus*	otu_3040	8	0.72	0.41	0.38	0.58	0.27	-0.03	-0.08	-0.08	0.28	0.42
Betaproteobacteria	*Comamonadaceae*	*Unclassified*	otu_3009	10	0.63	0.70	0.69	0.42	0.53	-0.15	-0.13	-0.13	0.10	-0.01
Flavobacteria	*Cryomorphaceae*	*Unclassified*	otu_1067	10	0.36	0.21	0.21	0.35	0.12	0.57	0.54	0.54	0.13	0.12
Gammaproteobacteria	*Aeromonadaceae*	*Aeromonas schubertii*	otu_3390	2	0.64	0.98	0.98	0.07	0.92	-0.17	-0.13	-0.13	-0.03	-0.13
Betaproteobacteria	*Gallionellaceae*	*Unclassified*	otu_3080	7	-0.08	-0.20	-0.21	0.88	-0.27	0.34	0.36	0.36	0.67	-0.05
Gammaproteobacteria	*Crenotrichaceae*	*Crenothrix sp.*	otu_3681	5	0.01	-0.24	-0.25	0.62	-0.31	0.66	0.65	0.65	0.42	0.10
Flavobacteria	*Flavobacteriaceae*	*Flavobacterium sp.*	otu_1102	10	0.25	0.30	0.30	0.81	0.23	-0.25	-0.22	-0.22	0.61	-0.12
Alphaproteobacteria	*Rhodobacteraceae*	*Unclassified*	otu_2666	10	0.67	0.98	0.98	-0.03	0.92	-0.10	-0.08	-0.08	-0.12	-0.11
Gammaproteobacteria	*Aeromonadaceae*	*Tolumonas sp.*	otu_3396	2	0.65	1.00	0.99	-0.01	0.94	-0.16	-0.12	-0.12	-0.10	-0.12
Betaproteobacteria	*Unassigned*	*Leptothrix discophora*	otu_2919	9	0.55	0.00	-0.01	0.60	-0.08	0.30	0.23	0.23	0.39	0.39
Sphingobacteria	*Flexibacteraceae*	*Unclassified*	otu_1205	8	0.64	0.86	0.84	0.09	0.74	-0.12	-0.06	-0.06	-0.15	-0.20
Betaproteobacteria	*Rhodocyclaceae*	*Unclassified*	otu_3146	10	0.02	-0.28	-0.30	0.79	-0.37	0.43	0.41	0.41	0.55	0.18
Actinobacteria	*Microbacteriaceae*	*Unclassified*	otu_441	10	0.30	-0.06	-0.07	0.80	-0.16	0.08	0.01	0.01	0.57	0.44
Sphingobacteria	*Flexibacteraceae*	*Emticicia sp.*	otu_1211	8	0.68	1.00	0.99	-0.08	0.94	-0.13	-0.10	-0.10	-0.17	-0.11
Betaproteobacteria	*Rhodocyclaceae*	*Methyloversatilis sp.*	otu_3158	4	-0.12	-0.21	-0.23	0.44	-0.30	0.76	0.81	0.81	0.25	-0.21
Gammaproteobacteria	*Unclassified*	*Unclassified*	otu_3370	6	0.65	1.00	1.00	-0.10	0.95	-0.15	-0.11	-0.11	-0.17	-0.11
Gammaproteobacteria	*Crenotrichaceae*	*Crenothrix polyspora*	otu_3682	3	0.45	0.64	0.68	-0.21	0.69	0.51	0.51	0.51	-0.14	-0.21
Betaproteobacteria	*Gallionellaceae*	*Gallionella sp.*	otu_3081	5	-0.11	-0.13	-0.14	0.99	-0.19	-0.05	-0.01	-0.01	0.82	-0.13
Sphingobacteria	*Flexibacteraceae*	*Flectobacillus sp.*	otu_1212	6	0.66	1.00	0.99	-0.12	0.94	-0.16	-0.12	-0.12	-0.20	-0.12
Betaproteobacteria	*Unclassified*	*Unclassified*	otu_3088	5	-0.18	-0.25	-0.27	0.61	-0.27	0.57	0.62	0.62	0.50	-0.25
Epsilonproteobacteria	*Campylobacteraceae*	*Arcobacter sp.*	otu_3314	4	-0.07	-0.17	-0.17	0.97	-0.22	0.02	0.05	0.05	0.81	-0.17
Alphaproteobacteria	*Sphingomonadaceae*	*Novosphingobium stygium*	otu_2886	3	0.44	0.83	0.81	-0.20	0.74	0.14	0.19	0.19	-0.35	-0.20

OTU	*Lepidocephalichthys sp.*	*Gallus gallus*	*Mastacembelus mastacembelus*	*Rattus sp.*	*Devario devario*	*Heteropneustes fossilis*	*Barbus eburneensis*	*Euphlyctis cyanophlyctis*	*Homo sapiens*	*Metahomaloptera omeiensis*
otu_1704	0.38	0.56	0.55	-0.81	0.50	0.65	0.69	0.69	-0.66	-0.18
otu_454	0.02	-0.21	-0.22	0.10	-0.26	0.98	0.98	0.98	-0.06	-0.03
otu_1229	0.28	-0.02	-0.03	0.80	-0.22	0.19	0.15	0.15	0.52	0.26
otu_3040	-0.07	0.43	0.43	0.17	0.40	0.59	0.67	0.67	0.12	-0.55
otu_3009	0.08	0.12	0.10	0.36	0.13	0.71	0.72	0.72	0.31	-0.03
otu_1067	0.37	0.62	0.59	0.42	0.54	-0.07	0.01	0.01	0.28	-0.17
otu_3390	0.13	-0.65	-0.64	0.65	-0.55	0.66	0.65	0.65	0.41	0.08
otu_3080	0.74	0.95	0.95	-0.16	0.89	0.21	0.22	0.22	-0.29	0.01
otu_3681	0.64	0.97	0.96	0.21	0.91	-0.10	-0.04	-0.04	0.03	-0.13
otu_1102	0.46	0.51	0.49	0.02	0.42	0.71	0.71	0.71	-0.17	0.06
otu_2666	0.13	-0.33	-0.47	0.70	-0.40	0.57	0.57	0.57	0.47	0.06
otu_3396	0.16	-0.68	-0.61	0.68	-0.48	0.61	0.61	0.61	0.45	0.06
otu_2919	0.25	0.73	0.71	0.24	0.65	0.25	0.33	0.33	0.06	-0.38
otu_1205	0.17	0.09	0.07	0.60	0.01	0.57	0.55	0.55	0.49	0.12
otu_3146	0.62	0.97	0.97	0.08	0.93	0.16	0.20	0.20	-0.08	-0.18
otu_441	0.42	0.76	0.75	0.09	0.70	0.41	0.48	0.48	-0.08	-0.41
otu_1211	0.16	-0.43	-0.40	0.71	-0.36	0.57	0.57	0.57	0.49	0.05
otu_3158	0.70	0.86	0.86	0.37	0.81	-0.13	-0.15	-0.15	0.18	0.11
otu_3370	0.20	-0.52	-0.30	0.71	-0.34	0.57	0.57	0.57	0.48	0.05
otu_3682	0.33	0.32	0.27	0.81	0.11	0.12	0.14	0.14	0.45	0.12
otu_3081	0.68	0.78	0.77	-0.37	0.70	0.48	0.49	0.49	-0.37	0.05
otu_1212	0.21	0.29	0.00	0.71	-0.23	0.56	0.56	0.56	0.49	0.05
otu_3088	0.73	0.87	0.87	0.28	0.76	0.09	0.08	0.08	0.01	0.13
otu_3314	0.64	0.80	0.78	0.00	0.71	0.43	0.43	0.43	-0.30	0.08
otu_2886	0.35	0.26	0.22	0.76	0.10	0.36	0.35	0.35	0.60	0.09

Supplementary Fig. S4
